# Recurrent Adrenergic Stress Provokes Persistent Myocarditis in PD-1–Deficient Mice

**DOI:** 10.1016/j.jacbts.2023.07.012

**Published:** 2023-09-20

**Authors:** Tomohiro Hayashi, Kenji Rowel Q. Lim, Attila Kovacs, Douglas L. Mann

**Affiliations:** a Center for Cardiovascular Research, Cardiovascular Division, Department of Medicine, Washington University School of Medicine, St. Louis, Missouri, USA; b Division of Community Medicine and Career Development, Kobe University Graduate School of Medicine, Kobe, Japan

**Keywords:** immune checkpoints, inflammation, myocarditis, tissue injury

## Abstract

It is unclear how the immune system initiates effective tissue repair responses without also simultaneously activating adaptive immune responses to self-antigens released by damaged or necrotic cells. We studied the role of repetitive adrenergic mediated stress on cardiac injury wild-type and programmed death-1–deficient (PD-1^−/−^) mice treated with 3 intraperitoneal low doses of isoproterenol followed by an intraperitoneal injection of high-dose ISO 7 days later (ISO^primed^/ISO^injury^). Repetitive adrenergic stress in ISO^primed^/ISO^injury^ PD-1^−/−^ mice resulted in a persistent dysregulated myocardial inflammatory response characterized by the expansion of autoreactive effector CD8^+^ T cells, increased cardiac hypertrophy, mild left ventricular dysfunction, and increased lethality when compared with ISO^primed^/ISO^injury^ wild-type mice.

## Highlights

• WT and PD-1^−/−^ mice were treated with 3 sequential IP priming doses of low-dose ISO followed by an IP injection of high-dose ISO 7 days later (ISO^primed^/ISO^injury^).
• Repetitive neurohormonal stress in ISO^primed^/ISO^injury^ PD-1^−/−^ mice led to a dysregulated persistent inflammatory response in the heart characterized by the expansion of autoreactive effector CD8^+^ T cells as well as increased cardiac hypertrophy, mild LV dysfunction, and increased lethality when compared with ISO^primed^/ISO^injury^ WT mice.
• The mediastinal lymph nodes draining the hearts of the PD-1^−/−^ mice were remarkable for having increased numbers of effector memory CD8^+^ T cells, whereas there was no expansion in the numbers of CD4^+^ or CD8^+^ effector or effector memory cells in the mediastinal lymph nodes of the WT mice.
• The effects of repetitive neurohormonal stress were tissue autonomous, insofar as there was no detectable inflammation or fibrosis in the lung, kidney, skeletal muscle or liver in ISO^primed^/ISO^injury^ in PD-1^−/−^ mice, and only a small increase in inflammation in the kidney detectable after 35 days.
• These studies suggest a critical role for the PD-1 signaling axis in negatively regulating the emergence of self-reactive CD8^+^ T cell responses following cardiac injury and may provide insights into the development of smoldering myocarditis in patients who are treated with immune checkpoint inhibitors.

## Visual Abstract

**Figure:**
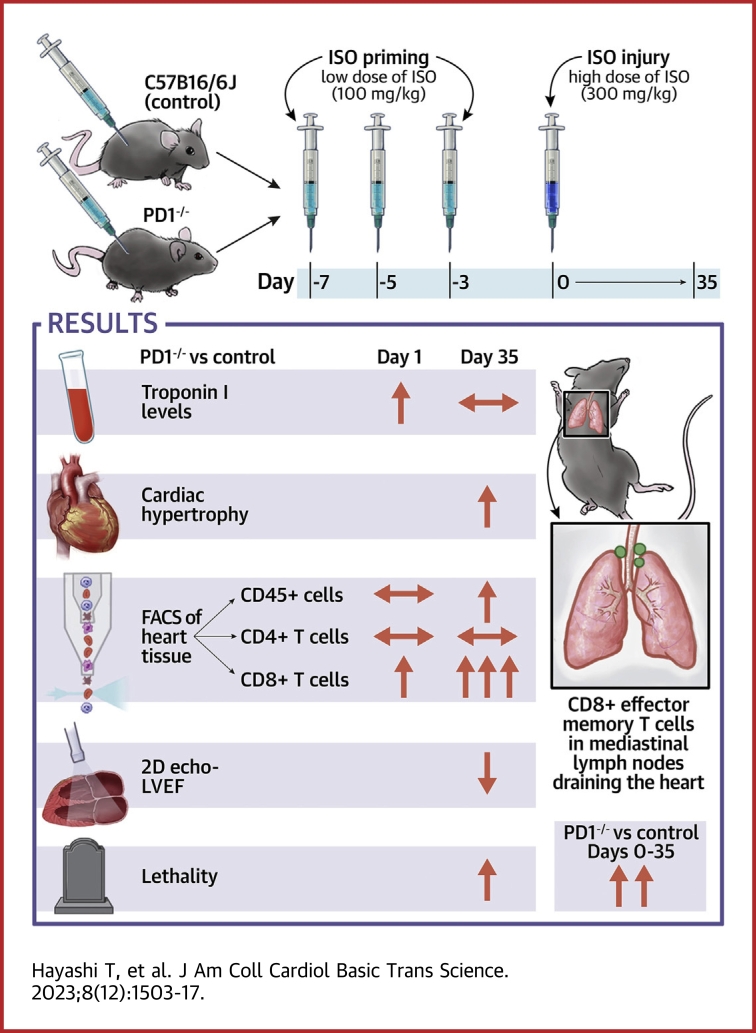

The immune system is essential for coordinating host responses to environmental danger, most notably through the detection and elimination of intruding pathogens,^1^ as well as by recruiting immune cells to sites of tissue injury to initiate tissue repair responses that are essential for maintaining host homeostasis and fitness.^2^, ^3^, ^4^ The ability of the immune system to simultaneously protect against both invading pathogens and orchestrate tissue repair processes is the result of evolutionary pressures that selected a diverse family of germ line–encoded innate immune receptors, which recognize shared molecular motifs that are common to both invading pathogens (referred to as pathogen-associated molecular patterns [PAMPs]) and to the molecular signals released by dying or dead/necrotic cells (referred to as damage-associated molecular patterns [DAMPs]). Once activated, these innate immune pattern recognition receptors initiate a sequence of highly optimized immune responses that preserve host survival and maintain host fitness.^1^^,^^3^^,^^5^

Although there has been extensive research with respect to the role of the innate and adaptive immune systems in reducing pathogen burden, far less is known about how immune mechanisms minimize the tissue damage attributable to invading pathogens or reduce the extent of collateral tissue damage caused by the brisk inflammatory response required to eliminate the pathogens.^1^^,^^5^^,^^6^ Moreover, the majority of studies to date have focused on understanding the role of immune responses to PAMPs associated with various pathogens,^5^, ^6^, ^7^ and have not as yet been extended to the much broader question of how the immune system initiates effective tissue repair responses following the release of DAMPs from host cells, without also simultaneously activating adaptive immune responses to previously concealed cytosolic and nuclear self-antigens released by damaged or necrotic cells.

Germane to this discussion, we have shown recently that transient myocardial injury induced by neurohormonal stress (isoproterenol [ISO]) provokes an acute inflammatory response in the heart that is accompanied by reversible changes in left ventricular (LV) structure and function.^8^ Remarkably, ISO-mediated neurohormonal stress provoked the tissue autonomous upregulation of co-inhibitory immune checkpoint receptors and ligands belonging to the programmed death-1 (PD-1): PD-ligand 1 (PD-L1) family in cardiac resident innate immune cells; whereas disrupting the PD-1:PD-L1 signaling axis resulted in a more exuberant and prolonged myocardial inflammatory response in ISO-treated mice that was associated with increased collateral tissue damage, delayed normalization of LV structure and function, and increased mortality.^8^ In subsequent studies, we demonstrated that ISO-mediated tissue injury and inflammation protected the heart from the myopathic effects of a recurrent challenge with ISO in WT mice, including decreased cardiac myocyte necrosis, reduced myocardial inflammation, preserved LV structure and function, and increased survival.^9^ Intriguingly, the ISO-mediated cytoprotective effects were abolished by depleting macrophages and dendritic cells using clodronate,^9^ suggesting that repetitive tissue injury leads to nonspecific innate immune cell–mediated protection against recurrent tissue injury (ie, trained innate immunity).^10^ To further explore the role of the PD-1 signaling axis in the context of recurrent myocardial tissue injury, we performed repetitive ISO-mediated neurohormonal stress in PD-1–deficient (PD-1^−/−^) mice. Here, we show that repetitive neurohormonal stress leads to a persistent dysregulated inflammatory response in the hearts of PD-1^−/−^ mice but not in the hearts of wild-type (WT) mice. The dysregulated immune response in PD-1^−/−^ mice was secondary to the clonal expansion of autoreactive cytotoxic effector CD8^+^ T cells that resulted in increased cardiac hypertrophy, persistent mild LV dysfunction, and increased mortality, thereby revealing a previously unappreciated role for the role of co-inhibitory immune checkpoints in restraining CD8^+^ T responses in the heart following repetitive tissue injury. Although speculative, these studies may also provide potential new mechanistic insights into the development of smoldering myocarditis in cancer patients who are being treated with immune checkpoint inhibitors (ICIs).^11^^,^^12^

## Methods

### Mice

C57BL/6J (stock no. 000664) and PD-1 receptor (PD-1^−/−^; C57BL/6J; B6.Cg-*Pdcd1*^*tm1.1Shr*^/J; stock no. 028276) mice were purchased from the Jackson Laboratory. The mouse colonies were maintained in a pathogen-free environment at the Washington University School of Medicine in St. Louis, Missouri, USA, and were fed pellet food and water ad libitum. We chose to study the *Pdcd1* deletion on a C57BL/6 background because these mice neither develop a cardiac phenotype nor experience increased mortality through 8 months of age.^13^ Because females were more likely to develop ICI-associated myocarditis based on clinical and preclinical studies,^14^, ^15^, ^16^ we predominately focused our studies on the response of 10-week-old female C57BL/6J mice and 10-week-old female PD-1^−/−^ mice. To determine whether there were sex-related differences, we repeated core experiments with 10-week-old male C57BL/6J mice and 10-week-old male PD-1^−/−^ mice.

### Study approval

All experimental procedures were performed in accordance with approved animal protocols from the Institutional Animal Care and Use Committee at Washington University School of Medicine in St. Louis, Missouri, USA. These investigations conform to the National Institutes of Health Guide for the Care and Use of Laboratory Animals.

### Induction of myocarditis in PD-1^−/−^ mice

**Figure 1:**
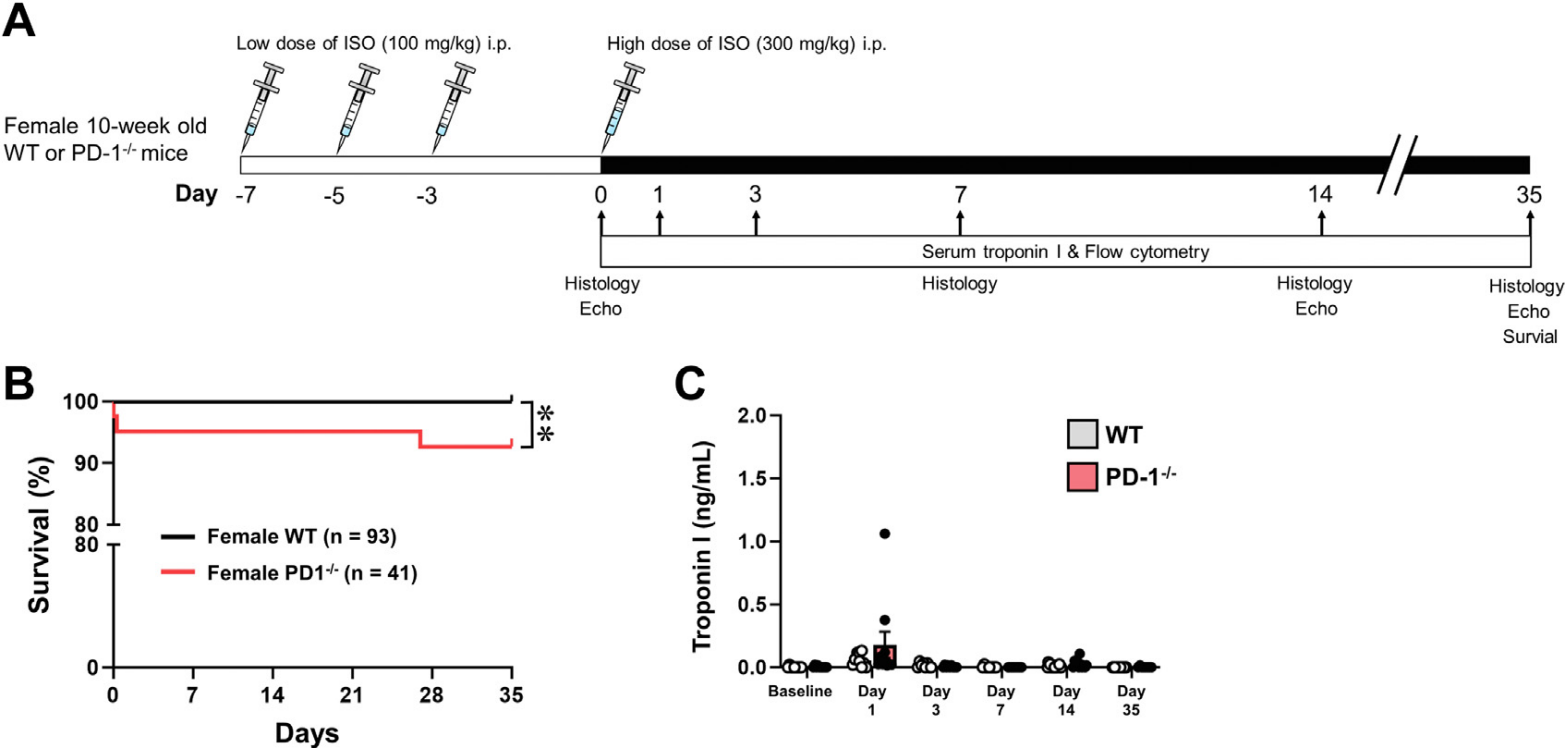
Study Protocol and Outcomes in ISO^primed^/ISO^injury^ WT and PD-1^−/−^ Mice (A) Study protocol and time course of evaluations for the 3 intraperitoneal low doses of isoproterenol (ISO^primed^) followed by an intraperitoneal injection of high-dose isoproterenol (ISO) 7 days later (ISO^primed^/ISO^injury^) experimental model. Mice were injected intraperitoneally (IP) with a low dose (100 mg/kg) of ISO on days −7, −5, and −3. On day 0 (baseline) the mice received a high dose (300 mg/kg) of ISO or diluent (phosphate buffered saline [PBS]) IP, and were evaluated at baseline (prior to high dose of ISO injection) and on days 1, 3, 7, 14, and 35. (B) Kaplan-Meier survival curves of female ISO^primed^/ISO^injury^ wild-type (WT) and programmed death 1–deficient (PD-1^−/−^) mice. (C) Serum troponin I levels at baseline and on days 1, 3, 7, 14, and 35 (n = 8-18 mice per group per time). Data were analyzed by repeated-measures 2-way analysis of variance with Sidak post hoc test. ∗*P* < 0.05; ∗∗*P* < 0.01.

The study protocol is illustrated in Figure 1A. PD-1^−/−^ or WT mice were injected intraperitoneally (IP) with 3 sequential priming doses of low-dose ISO (100 mg/kg) on days −7, −5, and −3, followed by a single high dose of ISO (300 mg/kg) on day 0 (baseline). ISO was dissolved in endotoxin-free phosphate-buffered saline (PBS) (Millipore; 0.05 mg/μL) and prepared on ice for immediate use as described previously.^8^

The doses of ISO that were used for these studies were based on prior ISO dose titration studies in C57BL/6J female mice (10 weeks of age), wherein we determined that a single IP injection of 100 mg/kg of ISO resulted in a numerically small increase in troponin I release that was not accompanied by myocardial inflammation,^8^ whereas a single IP injection of 300 mg/kg of ISO provoked a striking increase in troponin I release and brisk inflammatory response 24 hours after ISO injection. We have shown previously that a single ISO injection provokes a very low level of inflammation in the ISO-treated PD-1^−/−^ mice at day 7 after ISO, whereas there the number of CD8^+^ cells returned almost to baseline levels on day 7 in WT mice; however, the LV ejection fraction returned to baseline values by day 7 in the PD-1^−/−^ mice, indicating that the low levels of myocardial CD8^+^ cells observed after a single dose of ISO is not sufficient to lead to changes in LV function.^8^ In preliminary control experiments (Supplemental Figure 1A), we determined that there were numerically small but statistically nonsignificant increases (*P* = 0.25 by 2-way analysis of variance [ANOVA]) in troponin I release after the priming injections of 100 mg/kg of ISO in C57BL/6J (WT) and PD-1^−/−^ mice. As shown in Supplemental Figure 1B, 3 sequential low-dose ISO (100 mg/kg, IP) injections had no effect on survival in WT and PD-1^−/−^ mice.

### Serum troponin I levels

Serum troponin was measured using the ARCHITECT i2000 analyzer (Abbott Laboratories). Blood was collected by mandibular bleeding in BD Microtainer tubes at the time of terminal sacrifice. The serum was diluted 1:4 in PBS (80 μL serum + 240 μL PBS).

### Gravimetric and histological analysis

Mice were euthanized at baseline (prior to high-dose ISO injection) and on days 1, 3, 7, 14, and 35 after high-dose ISO injection and the hearts were removed and weighed to determine the heart weight-to-tibia length ratio as described.^17^ Tissues from the heart, skeletal muscle, lungs, spleen, and kidneys were processed, paraffin-embedded, and stained with hematoxylin and eosin and Masson’s trichrome. Additionally, hearts were stained with wheat germ agglutinin as described previously.^18^ The degree of inflammation in the heart and kidney was scored semi-quantitatively in the following manner using an inflammatory score index: 0 = no infiltrate; 1+ = infiltrates involving <25% of the ventricular myocardium/kidney; 2+ = infiltrates involving 25% to 50% of the myocardium/kidney; 3+ = infiltrates involving 50% to 75% of the myocardium/kidney; and 4+ = infiltrates involving 75% to 100% of the myocardium/kidney.^19^

### 2-dimensional echocardiographic studies

#### Image acquisition

Ultrasound examination of the cardiovascular system was performed using a speckle-based strain analysis package implemented in the Vevo 3100 system (VisualSonics), as described.^20^

#### Imaging protocol

Mice were imaged by echocardiography at baseline, 2 weeks, and 5 weeks after high-dose ISO injection to evaluate LV regional and global structure and function, as described.^8^ Low-dose Avertin (0.005 mL/g IP) was used for sedation for all imaging studies.

### Flow cytometry

#### Isolation of leukocytes

Single-cell suspensions of hearts, mediastinal lymph nodes, and the spleen were prepared and generated immediately before analysis by flow cytometry, exactly as previously described.^8^ Red blood cells were lysed using ACK lysing buffer (Gibco) for 15 minutes on ice, and the remaining cells were resuspended in 200 μL of fluorescence-activated cell sorting (FACS) buffer (PBS with 2% fetal bovine serum and 2 mM EDTA) and stained with conjugated antibodies (Supplemental Table 1) for 30 minutes at 4 °C and washed with FACS buffer before analysis. Intracellular staining of Foxp3 was performed using the Foxp3 staining buffer set (eBioscience), according to manufacturer’s instructions. Data were acquired using Becton Dickinson analyzers (BD X20 or LSRFortessa) at the Washington University School of Medicine in St. Louis Department of Pathology Flow Cytometry and Sorting Core facility. Compensation controls were generated using UltraComp eBeads (Invitrogen) and verified on single-color control samples obtained by staining primary splenocytes. The gating strategies for the leukocytes are summarized in Supplemental Figure 2. Cell cytometric data were analyzed by FlowJo software (TreeStar).

### Statistical analysis

All data are presented as mean ± SEM. The Shapiro-Wilk test was used to determine whether the data were normally distributed. The Kaplan-Meier survival curves were compared using the log-rank test. Statistical comparisons between 2 experimental groups were performed using 2-tailed Student’s *t* test. One- or 2-way repeated-measures ANOVA was used to adjust for correlation over time in the longitudinal analyses. One-way ANOVA with Dunnett’s multiple post hoc comparisons (multiple comparisons to a control) were performed for comparisons across >2 groups. For 2-way ANOVA, Sidak’s correction was used for post hoc comparisons. All data were analyzed using GraphPad Prism version 8. A *P* value <0.05 was considered statistically significant.

## Results

### Effect of ISO priming and ISO injury in WT and PD-1^−/−^ mice

**Figure 2:**
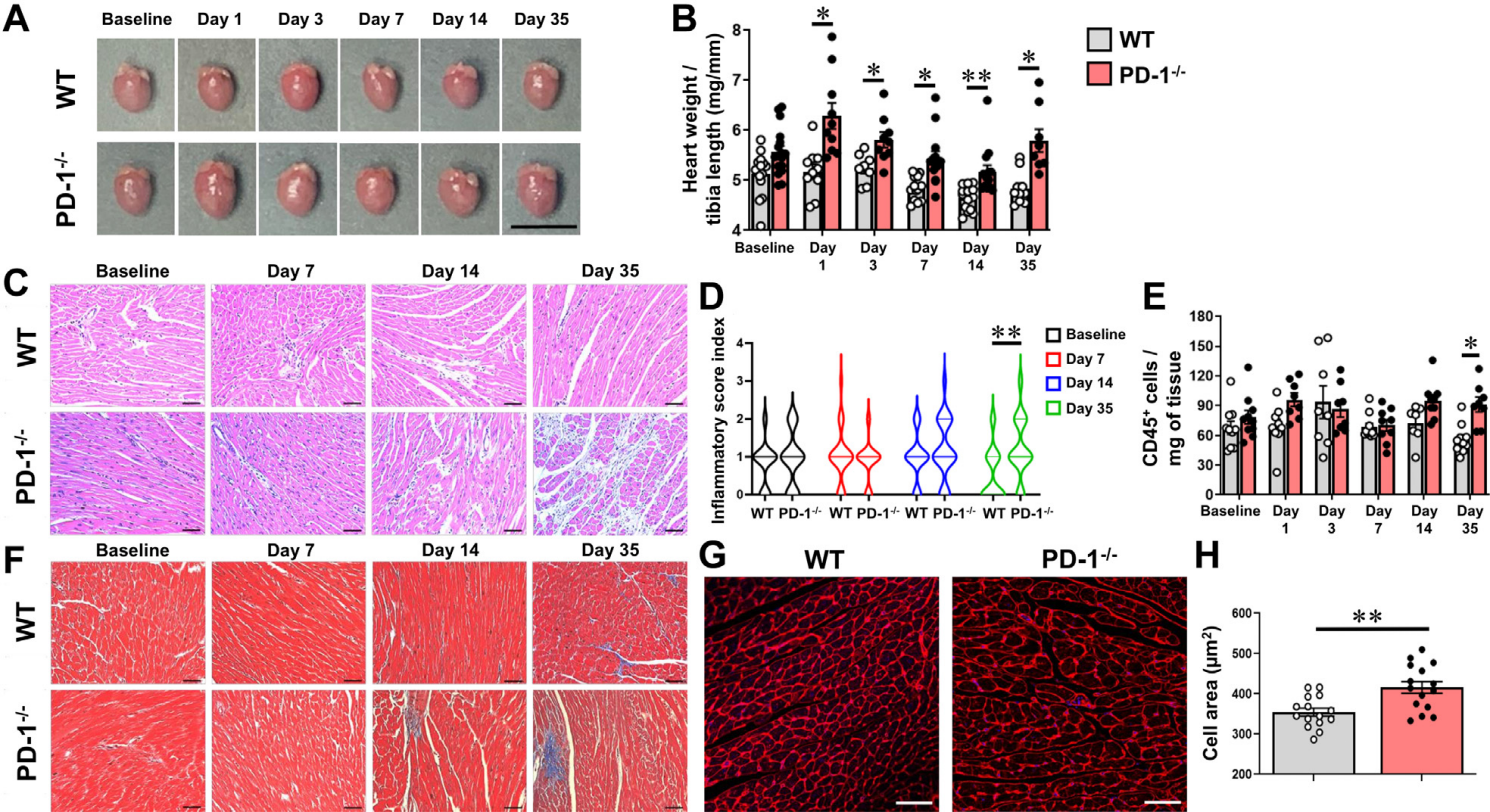
Characterization of ISO^primed^/ISO^injury^ WT and PD-1^−/−^ Mice (A) Representative photographs of ISO^primed^/ISO^injury^ whole hearts at baseline and on days 1, 3, 7, 14, and 35 (scale bar = 1 cm) after high-dose ISO injury. (B) Heart weight-to-tibia length ratios at baseline and on days 1, 3, 7, 14, and 35 (n = 8-17 mice per group per time). (C) Representative photomicrographs of hematoxylin and eosin–stained hearts at baseline and on days 7, 14, and 35 (scale bar = 50 μm). (D) Inflammatory score index at baseline and on days 7, 14, and 35 (n = 24-30 fields obtained from 4 to 5 hearts per group per time). The degree of inflammation in the heart was scored semi-quantitatively using an inflammatory score index: 0 = no infiltrate; 1+ = infiltrates involving <25% of the ventricular myocardium; 2+ = infiltrates involving 25% to 50% of the myocardium; 3+ = infiltrates involving 50% to 75% of the myocardium; and 4+ = infiltrates involving 75% to 100% of the myocardium.^19^ (E) Flow cytometry analysis of the number of CD45^+^ cells/mg of heart tissue at baseline and on days 1, 3, 7, 14, and 35 (n = 7-10 hearts per group per time). (F) Representative photographs of Masson’s trichrome–stained hearts at baseline and on days 7, 14, and 35 (scale bar = 50 μm). (G) Representative photographs of wheat germ agglutinin staining on day 35 (scale bar = 50 μm). (H) Group data of cardiac myocyte cell area determined by wheat germ agglutinin on day 35 (n = 20 cells counted per section, 3 sections per heart, 5 hearts per group). Data were analyzed by repeated-measures 2-way analysis of variance with Sidak post hoc test (B, D, E) or by 2-tailed Student's *t* test (H). ∗*P* < 0.05, ∗∗*P* < 0.01. Abbreviations as in Figure 1.

To determine the role of the PD-1 signaling axis in recurrent myocardial injury, we treated female WT and PD-1^−/−^ mice with 3 sequential antigen priming low doses of ISO (100 mg/kg [ISO^primed^]), followed by a single injection of high-dose ISO (300 mg/kg [ISO^injury^]) (see Figure 1A). As shown by the Kaplan Meier curves in Figure 1B, there were no deaths in the ISO^primed^/ISO^injury^ WT mice, consistent with our prior observations with ISO-induced preconditioning in WT mice.^9^ In sharp contrast, there was a statistically significant (*P* = 0.008) decrease in survival in the ISO^primed^/ISO^injury^ PD-1^−/−^ mice when compared with ISO^primed^/ISO^injury^ WT mice. Similar findings with respect to ISO-induced lethality were observed in male PD-1^−/−^ mice (Supplemental Figure 3A). There were no significant differences (*P* = 0.26 by 2-way ANOVA) in troponin I release in the WT and PD-1^−/−^ mice when followed for up to 35 days (Figure 1C). We did, however, note that there were significant increases in the heart weight-to-tibia length ratio in the ISO^primed^/ISO^injury^ PD-1^−/−^ mice compared with ISO^primed^/ISO^injury^ WT mice, which was detected as early as day 1 (*P* = 0.014) and persisted though day 3 (*P* = 0.048), day 7 (*P* = 0.012), day 14 (*P* = 0.007), and day 35 (*P* = 0.022) (Figures 2A and 2B). There were no changes in the heart weight-to-tibia length ratio in the ISO^primed^/ISO^injury^ WT mice compared with baseline, in agreement with our prior observations with ISO-induced preconditioning in WT mice.^9^

To characterize the myocardial inflammatory response in the ISO^primed^/ISO^injury^ mice, we performed histology and FACS in ISO^primed^/ISO^injury^ WT and PD-1^−/−^ mice. Figure 2C shows representative hematoxylin and eosin staining of leukocyte infiltrates at baseline and on days 7, 14, and 35 after high-dose ISO injection; group data are summarized in Figure 2D. The inflammatory score index was not significantly different in the ISO^primed^/ISO^injury^ WT and PD-1^−/−^ mice at baseline and on days 7 and 14; however, the inflammatory score index increased significantly (*P* = 0.001) in the ISO^primed^/ISO^injury^ PD-1^−/−^ mice on day 35 when compared with ISO^primed^/ISO^injury^ WT mice (Figure 2D). FACS revealed that there was a significant overall increase in the number of CD45^+^ leukocytes in hearts of ISO^primed^/ISO^injury^ PD-1^−/−^ mice relative to WT mice (*P* = 0.048 by 2-way ANOVA) and a significant increase (*P* = 0.011) in the number of CD45^+^ leukocytes in the hearts of ISO^primed^/ISO^injury^ PD-1^−/−^ mice on day 35 when compared with ISO^primed^/ISO^injury^ WT mice (Figure 2E). Masson’s trichrome staining revealed that there was increased patchy myocardial fibrosis that was more detectable in ISO^primed^/ISO^injury^ PD-1^−/−^ mice than in ISO^primed^/ISO^injury^ WT mice on day 35 (Figure 2F). We also examined myocyte cross-sectional area in the ISO^primed^/ISO^injury^ WT and PD-1^−/−^ mice on day 35. As shown by the representative wheat germ agglutinin staining of cardiac myocytes in cross-sections of ISO^primed^/ISO^injury^ WT and PD-1^−/−^ mouse hearts (Figure 2G) and the results of group data (Figure 2H), myocyte cross-sectional area was increased significantly (*P* = 0.002) in PD-1^−/−^ mice on day 35 when compared with WT mice, indicating that the increase in heart weight-to-tibia length ratio in the ISO^primed^/ISO^injury^ PD-1^−/−^ mice on day 35 was secondary, at least in part, to cardiac myocyte hypertrophy.

**Figure 3:**
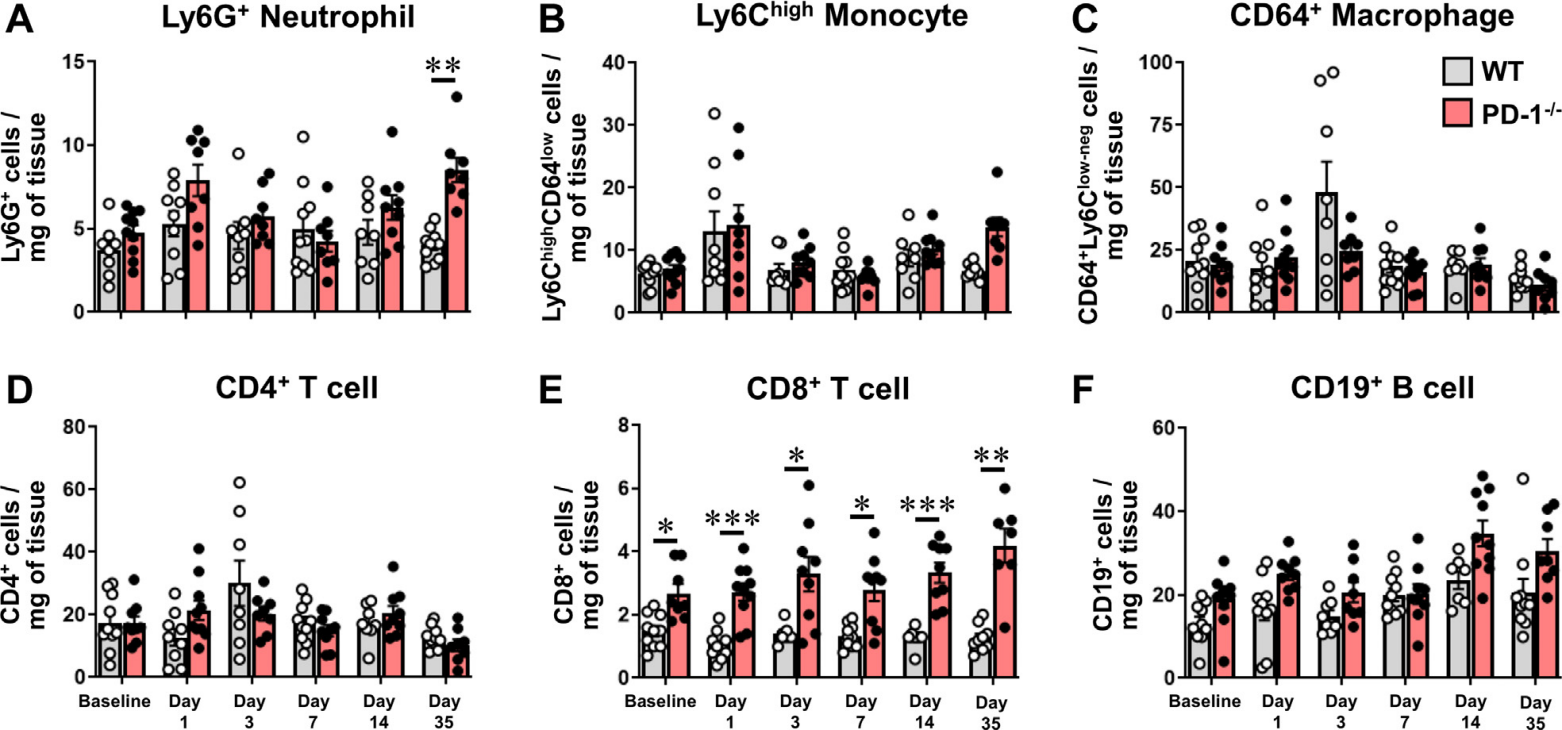
Immunophenotyping of Myocardial CD45^+^ Leukocytes in ISO^primed^/ISO^injury^ Mice Flow cytometric analyses of myocardial CD45^+^ cells (cells/mg of tissue) that were isolated from WT and PD-1^−/−^ mice at baseline and on days 1, 3, 7, 14, and 35 days after high-dose ISO injection (n = 7-10 mice per group per time). (A) Ly6G^+^ neutrophils. (B) Ly6C^high^CD64^low^ monocytes. (C) CD64^+^Ly6C^low/−^ macrophages. (D) CD4^+^ T cells. (E) CD8^+^ T cells. (F) CD19^+^ B cells. Data were analyzed by repeated-measures 2-way analysis of variance with Sidak post hoc test. ∗*P* < 0.05, ∗∗*P* < 0.01, ∗∗∗*P* < 0.001. Abbreviations as in Figure 1.

To explore the subsets of immune cells in the heart in ISO^primed^/ISO^injury^ WT and PD-1^−/−^ mice, we performed FACS at baseline and on days 1, 3, 7, 14, and 35 following the high-dose ISO injection. Relative to baseline values, there were no significant differences in the number of influxing neutrophils in the WT (*P* = 0.37) and PD-1^−/−^ mice (*P* = 0.071) on day 1 after the high-dose ISO injection, consistent with our prior observations with ISO-induced preconditioning (Figure 3A).^9^ However, on day 35, the number of Ly6G^+^ neutrophils in the ISO^primed^/ISO^injury^ PD-1^−/−^ mice was significantly increased (*P* = 0.002) relative to ISO^primed^/ISO^injury^ WT mice (Figure 3A). In contrast, there were no significant differences between ISO^primed^/ISO^injury^ WT and PD-1^−/−^ mice with respect to the number of Ly6C^high^CD64^low^ monocytes (*P* = 0.11 by 2-way ANOVA) (Figure 3B), CD64^+^Ly6C^low/−^ macrophages (*P* = 0.12 by 2-way ANOVA) (Figure 3C), CD4^+^ T cells (*P* = 0.76 by 2-way ANOVA) (Figure 3D), or CD19^+^ B cells (*P* = 0.19 by 2-way ANOVA) (Figure 3F), in accordance with our prior observations with ISO-induced preconditioning.^9^ However, the salient finding shown in Figure 3 is that loss of the PD-1 signaling axis resulted in a significant expansion in the number of CD8^+^ T cells, which was detectable at baseline (*P* = 0.029) after ISO-induced antigen priming, as well as on day 1 *(P* < 0.001), day 3 (*P* = 0.049), day 7 (*P* = 0.024), day 14 (*P* < 0.001), and day 35 (*P* = 0.007) (Figure 3E) after ISO injury. Similar overall results were obtained in ISO^primed^/ISO^injury^ male mice (Supplemental Figure 3). There were, however, several notable differences between male and female PD-1^−/−^ mice on day 35, including a significantly greater increase in the heart weight-to-tibia length ratio (*P* = 0.037) and a larger number of myocardial CD64^+^Ly6C^low/−^ macrophages (*P* = 0.012) and CD4^+^ T cells (*P* = 0.006) in the hearts of male mice.

The observation that the number of the CD8^+^ T cells was increased significantly at baseline (ie, prior to high-dose ISO injection) in the ISO^primed^ PD-1^−/−^ mice relative to ISO^primed^ WT mice raised the question of whether the phenotype in the ISO^primed^/ISO^injury^ PD-1^−/−^ mice, as well as the observed expansion of CD8^+^ T cells, was secondary to the sequential priming doses of low-dose ISO, which had negligible effects on myocardial tissue injury and troponin I release (Supplemental Figure 1), or whether instead the expansion of CD8^+^ T cells required both sequential ISO priming and high-dose ISO injury. To address this, we repeated several of the previous core experiments in PD-1^−/−^ mice that received 3 priming injections of low-dose ISO followed by an IP injection of PBS on day 0 (referred to as PD-1^−/−^ ISO^primed^/PBS mice) and then compared these responses with those of the ISO^primed^/ISO^injury^ PD-1^−/−^ mice shown in Figure 1, Figure 2, Figure 3. As shown in Supplemental Figure 4, the outcomes and phenotype of the PD1^−/−^ ISO^primed^/PBS mice were significantly different from that of ISO^primed^/ISO^injury^ PD-1^−/−^ mice, insofar as there were no deaths in the ISO^primed^/PBS mice, whereas there was a significant increase in lethality in the ISO^primed^/ISO^injury^ PD-1^−/−^ mice. Additionally, the heart-weight-to-tibia length ratio (*P* = 0.005), myocyte cell area (*P* = 0.036), and number of Ly6G^+^ neutrophils (*P* = 0.003) and CD8^+^ T lymphocytes (*P* = 0.018) were all significantly reduced on day 35 in the ISO^primed^/PBS PD-1^−/−^ mice when compared with the ISO^primed^/ISO^injury^ PD-1^−/−^ mice. Viewed together, these data suggest that the expansion of CD8^+^ T cells in the ISO^primed^/ISO^injury^ PD-1^−/−^ mice required both sequential ISO-induced antigen priming and high-dose ISO injury. Moreover, the previous results suggest that loss of PD-1^−/−^ receptor immune checkpoint signaling in the context of repetitive ISO-mediated injury results in the expansion of cytotoxic CD8^+^ lymphocytes in the heart, consistent with the development of “smoldering” CD8^+^ lymphocytic myocarditis that has been reported recently in cancer patients who are being treated with ICIs.^12^^,^^21^

Given the central role of conventional dendritic cells (cDCs) in CD8^+^ T cell proliferation and expansion,^22^ we also examined the major resident cDC subsets^22^ in the hearts of ISO^primed^/ISO^injury^ WT and PD-1^−/−^ mice at baseline and on days 7, 14, and 35. The gating strategies for these studies are shown in Supplemental Figure 5A. There were no significant differences between ISO^primed^/ISO^injury^ WT and PD-1^−/−^ mice with respect to the number of CD64^−^CD11c^+^MHC-II^high^ dendritic cells (*P* = 0.58 by 2-way ANOVA), nor in the number of CD103^+^CD11b^−^ ([cDC1] *P* = 0.89 by 2-way ANOVA) or CD11b^+^CD103^−^ cells ([cDC2] *P* = 0.52 by 2-way ANOVA) at all of the time points examined (Supplemental Figures 5B to 5D).^22^

### Characterization of CD8^+^ lymphocytic myocarditis in PD-1^−/−^ mice

**Figure 4:**
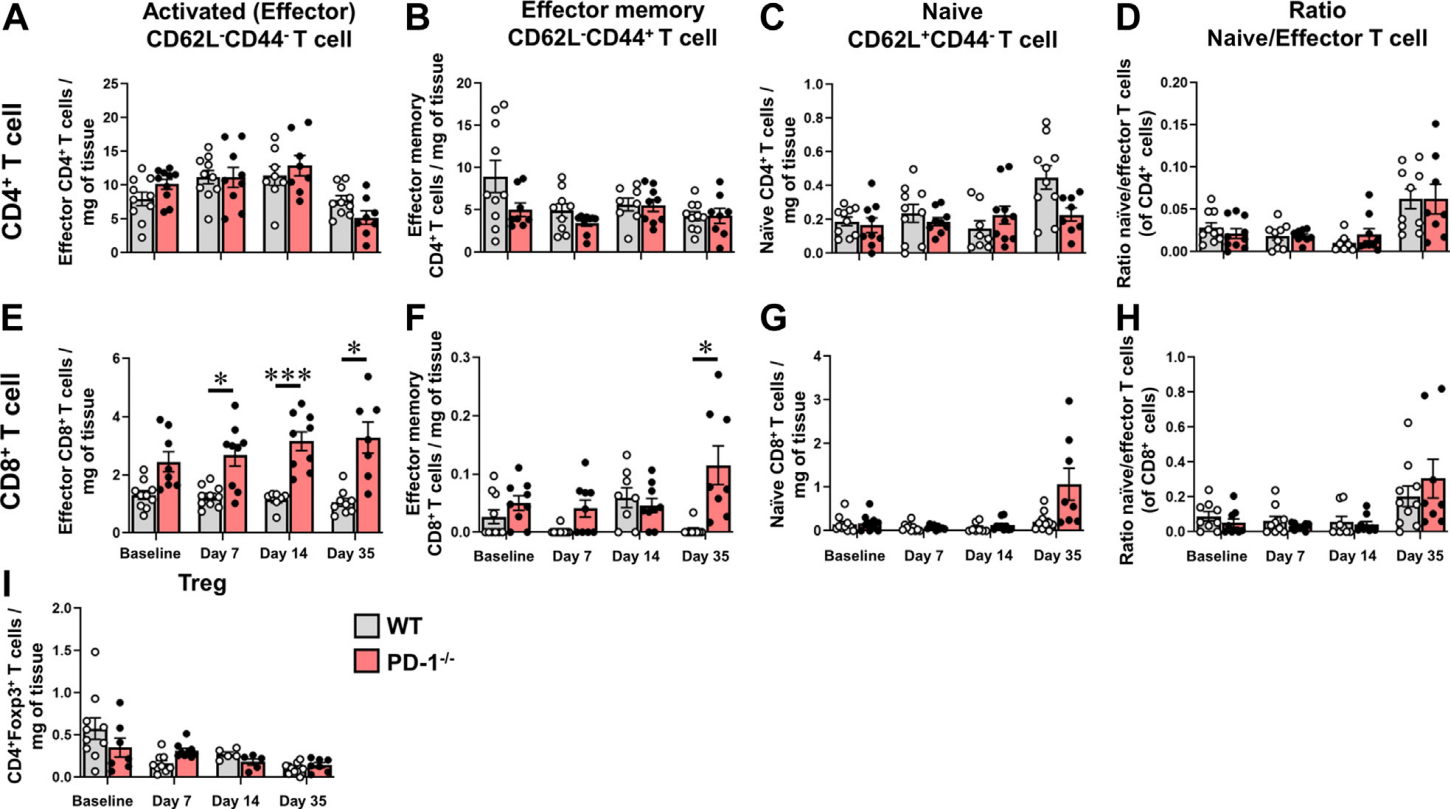
T Cell Subsets in the in the Hearts of ISO^primed^/ISO^injury^ Mice Flow cytometric analyses were performed on CD45^+^ cells (cells/mg of tissue) isolated from the hearts of WT and PD-1^−/−^ mice at baseline and on days 7, 14, and 35 days after ISO injection. (A) CD4^+^CD62L^−^CD44^−^ activated (effector) T cells, (B) CD4^+^CD62L^−^CD44^+^ effector memory T cells. (C) CD4^+^CD62L^+^CD44^−^ naive T cells, (D) Ratio of naive to effector CD4^+^ T cells, (E) CD8^+^CD62L^−^CD44^−^ activated (effector) T cells, (F) CD8^+^CD62L^−^CD44^+^ effector memory T cells. (G) CD8^+^CD62L^+^CD44^−^ naive T cells, (H) Ratio of naive to effector CD8^+^ T cells (n = 7-10 mice per group per time for A to H), (I) CD4^+^Foxp3^+^ regulatory T (Treg) cells (n = 5-11 mice per group per time). Data were analyzed by repeated-measures 2-way analysis of variance with Sidak post hoc test. ∗*P* < 0.05, ∗∗∗*P* < 0.001. Abbreviations as in Figure 1.

To further characterize the lymphocytic myocarditis in the ISO^primed^/ISO^injury^ PD-1^−/−^ mice, we examined subsets of myocardial T cells using CD62L and CD44 markers to differentiate among naïve, effector, and memory T cells. The gating strategy for these studies is illustrated in Supplemental Figure 2. As shown in Figures 4E to 4H, when compared with ISO^primed^/ISO^injury^ WT mice, there was a significant increase in the number of CD8^+^CD62L^−^CD44^−^ effector T cells in the ISO^primed^/ISO^injury^ PD1^−/−^ mice on days 7 (*P* = 0.018), 14 (*P* < 0.001), and 35 (*P* = 0.021) and a significant increase (*P* = 0.048) in the number of CD8^+^CD62L^−^CD44^+^ effector memory T cells on day 35 in the ISO^primed^/ISO^injury^ PD-1^−/−^ mice. In contrast, there were no significant differences in CD4^+^CD62L^−^CD44^−^ effector (*P* = 0.72 by 2-way ANOVA) or CD4^+^CD62L^−^CD44^+^ effector memory (*P* = 0.085 by 2-way ANOVA) T cells between the ISO^primed^/ISO^injury^ WT and PD-1^−/−^ mice at any time point examined (Figures 4A to 4D). Importantly, the number of CD4^+^Foxp3^+^ regulatory T cells was not significantly different (*P* = 0.59 by 2-way ANOVA) between the ISO^primed^/ISO^injury^ WT and PD-1^−/−^ mice over the time course of the study (Figure 4I). Viewed together, these results indicate that low-dose ISO priming followed by high-dose ISO injury leads to activation of CD8^+^CD62L^−^CD44^−^ effector T cells in PD1^−/−^ mice, whereas T cell activation does not occur in WT mice with an intact PD-1 immune checkpoint signaling axis.

**Figure 5:**
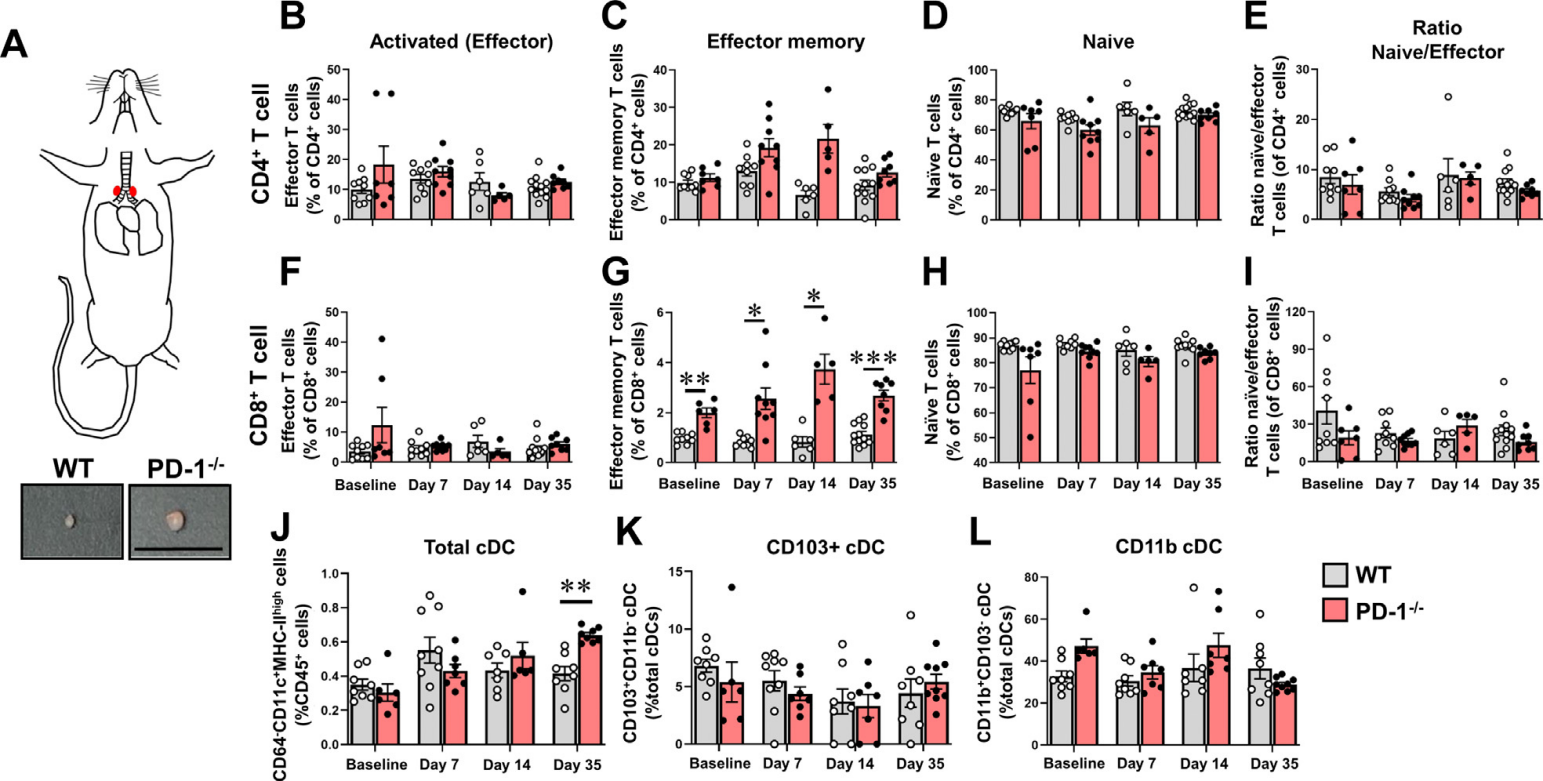
T Cell and cDC Subsets in Mediastinal Lymph Nodes of ISO^primed^/ISO^injury^ Mice Flow cytometric analyses were performed on CD4^+^ T cells, CD8^+^ T cells, and conventional dendritic cells (cDCs) isolated from the mediastinal lymph nodes of WT and PD-1^−/−^ mice at baseline and on days 7, 14, and 35 days after ISO injection. (A) Illustration of the anatomic location of mediastinal lymph nodes (shown in red) and photograph of mediastinal lymph nodes used for analyses (scale bar = 1 cm). (B) CD4^+^CD62L^−^CD44^−^ activated (effector) T cells (% of total CD4^+^ T cells). (C) CD4^+^CD62L^−^CD44^+^ effector memory T cells (% of total CD4^+^ T cells). (D) CD4^+^CD62L^+^CD44^−^ naive T cells (% of total CD4^+^ T cells). (E) Ratio of naive to effector CD4^+^ T cells. (F) CD8^+^CD62L^−^CD44^−^ activated (effector) T cells (% of total CD8^+^ T cells). (G) CD8^+^CD62L^−^CD44^+^ effector memory T cells (% of total CD8^+^ T cells). (H) CD8^+^CD62L^+^CD44^−^ naive T cells (% of total CD8^+^ T cells). (I) Ratio of naive to effector CD8^+^ T cells (n = 5-12 mice per group per time in B to I). (J) Total number of cDCs identified as CD64^−^CD11c^+^MHC-II^high^ cells, expressed as a % of CD45^+^ cells, as well as number of (K) CD103^+^ cDCs and (L) CD11b^+^ cDCs (n = 6-10 mice per group per time B to L). Data were analyzed by repeated-measures 2-way analysis of variance with Sidak post hoc test. ∗*P* < 0.05, ∗∗*P* < 0.01, ∗∗∗*P* < 0.001. Abbreviations as in Figure 1.

Activation of a T cell response requires antigen presentation to T cells, which typically occurs in the respective draining lymph nodes, rather than directly at the sites of tissue injury. Prior studies in mice have shown that lymph nodes in the upper mediastinum are responsible for draining the heart following necrotic myocyte injury, whereas cervical and inguinal lymph nodes, spleen, and bone marrow are not involved.^23^ Accordingly, we isolated cells from the draining mediastinal lymph nodes of ISO^primed^/ISO^injury^ WT and PD-1^−/−^ mice at baseline, and on days 7, 14, and 35, and then performed FACS to in order to examine the CD4^+^ and CD8^+^ T cell subsets, and cDCs at the site (gating strategy shown in Supplemental Figure 6). Qualitative examination of the mediastinal lymph nodes from ISO^primed^/ISO^injury^ PD-1^−/−^ mice revealed that there was a ∼2-fold increase in their size compared with that in ISO^primed^/ISO^injury^ WT mice (Figure 5A). FACS revealed that there were no significant differences between ISO^primed^/ISO^injury^ WT and PD-1^−/−^ mice with respect to the number of CD4^+^CD62L^−^CD44^−^ effector T cells (*P* = 0.41 by 2-way ANOVA) (Figure 5B) or CD4^+^CD62L^−^CD44^+^ effector memory T cells (*P* = 0.17 by 2-way ANOVA) (Figure 5C), which is internally consistent with the observation that there were no differences in CD4^+^ T cell subsets between the hearts of ISO^primed^/ISO^injury^ WT and PD-1^−/−^ mice (Figures 3D and 4A to 4D). Although there were no significant differences between PD-1^−/−^ and WT mice with respect to the number of CD8^+^CD62L^−^CD44^−^ effector T cells (*P* = 0.23 by 2-way ANOVA) (Figure 5F), there was a significant increase in the number of CD8^+^CD62L^−^CD44^+^ effector memory T cells in the mediastinal lymph nodes from PD-1^−/−^ mice relative to WT mice at baseline (*P* = 0.009), as well as on days 7 (*P* = 0.015), 14 (*P* = 0.024), and 35 (*P* < 0.001) (Figure 5G), suggesting that antigen presentation and CD8^+^ T cell activation occurred in the mediastinal lymph nodes draining the hearts of ISO^primed^/ISO^injury^ PD-1^−/−^ mice. Consistent with this observation, we also observed a significant overall difference (*P* = 0.002 by 2-way ANOVA) in the number of total cDCs in the mediastinal lymph nodes of the ISO^primed^/ISO^injury^ PD-1^−/−^ mice relative to ISO^primed^/ISO^injury^ WT mice (Figure 5J). However, there were no significant differences in the subsets of cDCs between the WT and PD-1^−/−^ mice at any time point examined (Figures 5K and 5L).

**Figure 6:**
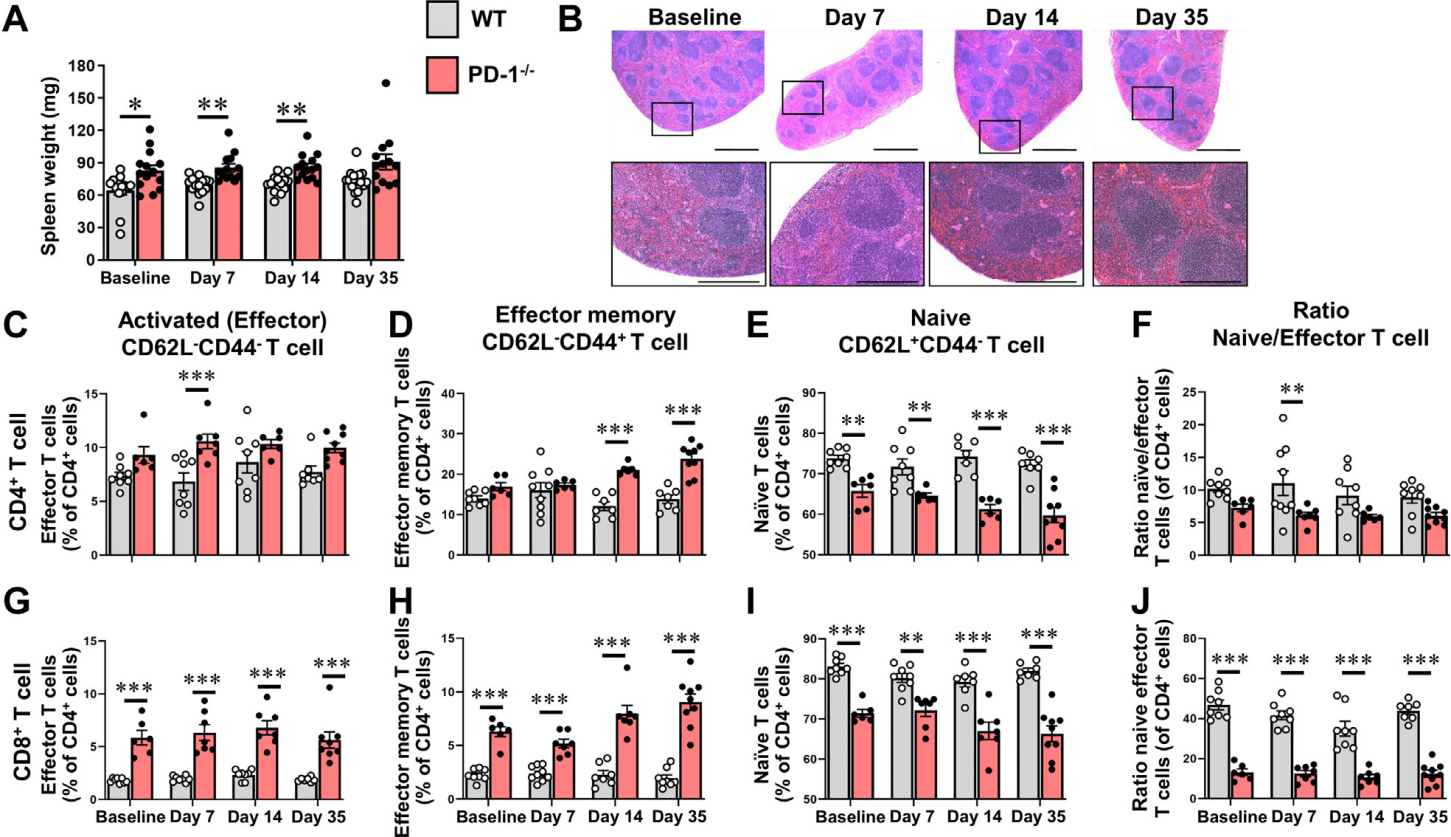
Splenic Remodeling and T Cell Subsets in the Spleens of ISO^primed^/ISO^injury^ Mice (A) Spleen weights at baseline and on days 1, 3, 7, 14, and 35 (n = 8-17 mice per group per time) in the ISO^primed^/ISO^injury^ WT and PD-1^−/−^ mice. (B) Representative photomicrographs of hematoxylin and eosin–stained spleens at baseline and on days 7, 14, and 35 days in ISO^primed^/ISO^injury^ PD-1^−/−^ mice (scale bar = 1 mm), with representative high-power fields (scale bar = 500 μm) of cropped hematoxylin and eosin–stained sections shown (bottom). (C) CD4^+^CD62L^−^CD44^−^ activated (effector) T cells (% of total CD4^+^ T cells). (D) CD4^+^CD62L^−^CD44^+^ effector memory T cells (% of total CD4^+^ T cells). (E) CD4^+^CD62L^+^CD44^−^ naive T cells (% of total CD4^+^ T cells). (F) Ratio of naive to effector CD4^+^ T cells, (G) CD8^+^CD62L^−^CD44^−^ activated (effector) T cells (% of total CD8^+^ T cells). (H) CD8^+^CD62L^−^CD44^+^ effector memory T cells (% of total CD8^+^ T cells). (I) CD8^+^CD62L^+^CD44^−^ naive T cells (% of total CD8^+^ T cells). (J) Ratio of naive/effector CD8^+^ T cells (n = 6-17 mice per group per time A and C to J). Data were analyzed by repeated-measures 2-way analysis of variance with Sidak post hoc test. ∗*P* < 0.05, ∗∗*P* < 0.01, ∗∗∗*P* < 0.001. Abbreviations as in Figure 1.

We also observed a significant increase in splenic weight in the ISO^primed^/ISO^injury^ PD-1^−/−^ mice relative to WT mice (Figure 6A) that was detected at baseline (*P* = 0.033) and on days 7 (*P* = 0.004), 14 (*P* = 0.002), and 35 (*P* = 0.051), as well as a qualitative increase in the number and size of the white pulp follicles on days 14 and 35 (Figure 6B), suggestive of heightened antigen processing in the spleens of the ISO^primed^/ISO^injury^ PD-1^−/−^ mice. To characterize the CD4^+^ and CD8^+^ cell subsets in the spleen, we performed FACS analysis of total splenic extracts obtained from ISO^primed^/ISO^injury^ WT and PD-1^−/−^ mice at baseline and on day 7, 14, and 35. The gating strategy for these studies is illustrated in Supplemental Figure 6. As shown in Figure 6C, there was a significant increase in the number of CD4^+^CD62L^−^CD44^−^ effector T cells in the spleens of the PD-1^−/−^ mice on day 7 (*P* < 0.001) (Figure 6C) and a significant increase in the number of splenic CD4^+^CD62L^−^CD44^+^ effector memory T cells on days 14 and 35 (Figure 6D) (*P* < 0.001 for both), that was accompanied by a significant decrease in the number of naive CD4+CD62L+CD44+ T cells on days 7 (*P* = 0.007), 14 (*P* < 0.001), and 35 (*P* < 0.001). Consistent with our observations with respect to the presence of a CD8^+^ T cell–dominant myocarditis in the hearts of the ISO^primed^/ISO^injury^ PD-1^−/−^ mice, we detected significant differences in the number of CD8^+^CD62L^−^CD44^−^ effector T cells (Figure 6G) and CD8^+^CD62L^−^CD44^+^ effector memory T cells (Figure 6H), as well as a significant decrease in the ratio of naïve/effector CD8^+^ T cells (Figure 6J) in the spleen at all the time points that were examined. Viewed together, these results are consistent with cDC migration to the draining lymph nodes following ISO injury, particularly with subsequent activation and expansion of CD8^+^ T cell clones in the draining mediastinal lymph nodes and in the white pulp of the spleens of ISO^primed^/ISO^injury^ PD-1^−/−^ mice.

### Functional significance of CD8^+^ lymphocytic myocarditis in PD-1^−/−^ mice

**Figure 7:**
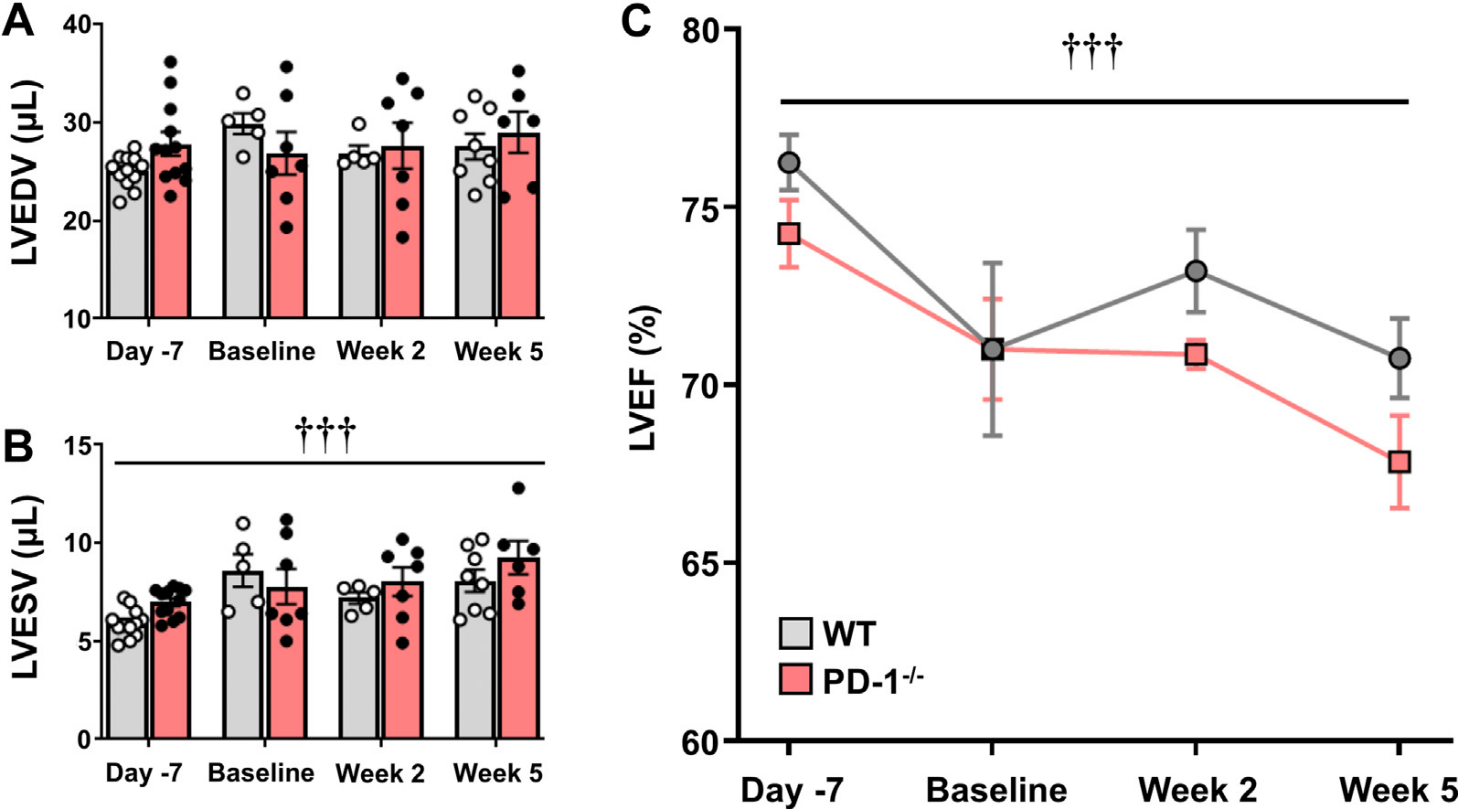
Two-Dimensional Echocardiographic Assessment of Left Ventricular Structure and Function ISO^primed^/ISO^injury^ Mice (A) Left ventricular end-diastolic volume (LVEDV), (B) left ventricular end-systolic volume (LVESV), and (C) left ventricular ejection fraction (LVEF) in naivenaive female WT and PD-1^−/−^ mice, WT and PD-1^−/−^ mice at baseline (ISO^primed^), and ISO^primed^/ISO^injury^ WT and PD-1^−/−^ mice at weeks 2 and 5 (n = 5-12 mice per group per time). Data were analyzed by repeated-measures 2-way analysis of variance with Sidak post hoc test. †††*P* < 0.001 by repeated-measures 2-way analysis of variance. Abbreviations as in Figure 1.

To ascertain whether the persistent cytotoxic CD8^+^ lymphocytic infiltrates in the hearts of the PD-1^−/−^ mice resulted in changes in LV structure and function, we performed 2-dimensional echocardiography in naive WT and PD-1^−/−^ mice, as well as in ISO^primed^ and ISO^primed^/ISO^injury^ WT and PD-1^−/−^ mice at baseline and at 2 and 5 weeks after the high-dose ISO injection (Figure 7). There was no significant overall difference in LV end-diastolic volume (*P* = 0.31 by 2-way ANOVA) in the WT and PD-1^−/−^ hearts following high-dose ISO injection (Figure 7A). However, there was a significant increase in LV end-systolic volume (*P* < 0.001 by 2-way ANOVA) and a progressive small but statistically significant decline in LV ejection fraction (*P* < 0.001 by 2-way ANOVA) in the ISO^primed^/ISO^injury^ PD-1^−/−^ mice when compared with ISO^primed^/ISO^injury^ WT mice (Figures 7B and 7C, respectively), suggesting that the low level persistent cytotoxic CD8^+^ lymphocytic myocarditis was functionally significant.

### Assessment of multiorgan involvement following ISO priming and ISO injury in PD-1^−/−^ mice

Recognizing that multiorgan involvement has been reported in cases of ICI-induced myocarditis,^24^ we also performed a histopathological examination of the lungs, kidney, skeletal muscle, and liver in ISO^primed^/ISO^injury^ female PD-1^−/−^ mice at baseline and on days 7, 14, and 35 after high-dose ISO injection. As shown in Supplemental Figure 7, there was no evidence of inflammation or fibrosis in the lung, kidney, skeletal muscle, or liver at baseline or on days 7 and 14, suggesting that the observed CD8^+^ myocarditis was secondary to the clonal expansion of autoreactive CD8^+^ cells. We did, however, observe a significant increase in inflammatory cell infiltrates in the kidney on day 35 (Supplemental Figure 7C), consistent with the off-target effects of ISO that have been reported in WT mice.^25^

## Discussion

Here, we show that repetitive neurohormonal stress in PD-1^−/−^ mice leads to a persistent dysregulated inflammatory response in the heart characterized by the expansion of autoreactive cytotoxic CD8^+^ T cells, increased cardiac hypertrophy, mild LV dysfunction, and increased lethality when compared with WT mice. Viewed together, these studies reveal a critical role for the PD-1 signaling axis in negatively regulating self-reactive CD8^+^ T cell responses following cardiac injury. The following 3 lines of evidence support these statements.

First, when PD-1^−/−^ mice were exposed to 3 priming doses of low-dose ISO (ISO^primed^) followed by an injection of high-dose ISO (ISO^primed^/ISO^injury^) there was an increase in mortality, whereas there were no deaths in ISO^primed^/ISO^injury^ WT mice (Figure 1B, Supplemental Figure 3A). Intriguingly, the increase in lethality in PD-1^−/−^ mice was only observed in ISO^primed^/ISO^injury^ PD-1^−/−^ mice, whereas there were no deaths in the ISO^primed^ PD-1^−/−^ mice (Supplemental Figure 1B), suggesting that prior myocardial injury is required but is not sufficient for the observed increase in mortality. Second, the myocardial inflammatory response in ISO^primed^/ISO^injury^ PD-1^−/−^ mice occurred early after ISO priming and was sustained after high-dose ISO injury (Figures 2C to 2E), whereas there was no myocardial inflammation observed in the ISO^primed^/ISO^injury^ WT mice (Figures 2C to 2E), consistent with our prior observations in this model.^9^ The persistent dysregulated myocardial inflammatory response in the ISO^primed^/ISO^injury^ PD-1^−/−^ mice was characterized by increased numbers of effector CD8^+^ T cells on days 1, 3, 7, 14, and 35, and a secondary increase in Ly6G^+^ neutrophils on day 35 (Figures 3 and 4). We also observed increased numbers of effector memory CD8^+^ T cells at baseline and on days 7, 14, and 35 in the mediastinal lymph nodes draining the hearts of the ISO^primed^/ISO^injury^ PD-1^−/−^ mice, whereas there was no expansion in the numbers of CD4^+^ or CD8^+^ effector or effector memory cells in the mediastinal lymph nodes of the ISO^primed^/ISO^injury^ WT mice (Figure 5). There was also significant splenic remodeling and an increase in the weight of the spleen and a qualitative increase in number and size of the white pulp follicles in the ISO^primed^/ISO^injury^ PD-1^−/−^ mice, as well as an increase in effector and effector memory CD8^+^ cells in the spleen (Figure 6), suggesting that PD-1 is important in regulating CD8^+^ T cell expansion in secondary lymphoid tissues. Remarkably, the ISO injury–induced clonal expansion of memory CD8^+^ T cells in PD-1^−/−^ mice was specific to the heart, insofar as there was no evidence of inflammation or fibrosis in the lung, kidney, skeletal muscle, or liver at baseline or on days 7 and 14, and there was only a small increase in inflammatory cell infiltrates in the kidney that was detectable on day 35 (Supplemental Figure 7C).

### Immune checkpoints and myocardial inflammation following tissue injury

Previously, we showed that a single injection of ISO provokes an acute self-limited myocardial inflammatory response that was accompanied by reversible changes in LV structure and function.^8^ Disruption of the PD-1/PD-L1/PD-L2 signaling axis resulted in a prolonged ISO-induced myocardial inflammatory response, which led to increased collateral tissue damage and a delay in the normalization of LV structure and function.^8^ These acute studies revealed a previously unappreciated role for the PD-1/PD-L1/PD-L2 signaling axis in regulating innate immune responses to tissue injury, insofar as the activation of CD4^+^ or CD8^+^ T cells was not required for the increased inflammatory response. Here, we expand on these initial findings by demonstrating that repetitive neurohormonal stress in PD-1^−/−^ mice leads to the expansion of autoreactive CD8^+^ T cells, sustained low-grade “smoldering myocarditis,” increased cardiac hypertrophy, irreversible LV dysfunction, and increased lethality. We also observed a significant increase in the heart weight-to-tibia length ratio and greater numbers of myocardial CD64^+^Ly6C^low/−^ macrophages and CD4^+^ T cells in the hearts of male PD1^−/−^ male mice when compared with female PD1^−/−^ mice.^26^ Viewed together, our past^8^^,^^27^ and current work highlights the remarkable complexity of the PD-1:PD-L1:PD-L2 signaling axis in regulating the myocardial inflammatory response in the setting of both acute and recurrent tissue injury.

Although this study was not specifically designed to elucidate the molecular mechanisms that were responsible for the clonal expansion of autoreactive cytotoxic CD8^+^ T cells in the hearts of the ISO^primed^/ISO^injury^ PD1^−/−^mice, there are several possible mechanisms that warrant discussion. Axelrod et al^28^ demonstrated the presence of clonal effector CD8^+^ T populations in the hearts of *Pdc1*^−/−^/*Ctla4*^+/−^ mice that develop spontaneous myocarditis. These authors performed T cell receptor (TCR) sequencing and identified 3 major histocompatibility complex I restricted TCRs specific for a cardiac specific protein (α-myosin) that was not detected in the thymus, suggesting that escape from central tolerance was responsible for the development of spontaneous CD8^+^ myocarditis in the *Pdc1*^−/−^/*Ctla4*^+/−^ mice. This study further showed that treatment with an anti-CD8 depleting antibody, but not an anti-CD4 depleting antibody improved survival in *Pdc1*^−/−^/*Ctla4*^+/−^ mice. Further, adoptive transfer of CD8^+^ lymphocytes from *Pdc1*^*−/−*^*/Ctla4*^*+/−*^ mice with myocarditis provoked fatal myocarditis in WT recipient mice, whereas adoptive transfer of CD4^+^ cells from *Pdc1*^*−/−*^*/Ctla4*^*+/−*^ mice had no effect.^28^ A second possible mechanism is that loss of peripheral immune tolerance in the PD-1^−/−^ mice resulted in the expansion of CD8^+^ lymphocytes that have low affinity TCRs for self-antigens and normally escape clonal deletion in the thymus.^29^^,^^30^ When these self-reactive T cells encounter self-antigens, they are inactivated in peripheral tissues because TCR ligation in the absence of positive costimulatory signals by antigen-presenting cells leads to functional inactivation of T cells (ie, anergy). Relevant to this discussion, the interaction of PD-1 with its 2 cognate ligands, PD-L1 and PD-L2, has been shown to inhibit T cell effector function in an antigen-specific manner, by providing negative costimulatory signals. Additionally, the PD-1/PD-L1/PD-L2 signaling axis has been shown to limit the initial phase of activation and expansion of self-reactive T cells,^31^ as well as regulate dynamic T cell mobility.^32^ We recently demonstrated that ISO injury provokes the tissue autonomous upregulation of the PD-1/PD-L1/PD-L2 signaling axis in immune cells in the heart and that the presence of intact PD-1/PD-L1/PD-L2 signaling axis attenuates the expansion of autoreactive myocardial CD8^+^ T cell populations following ISO-injury.^8^ A third possibility is that dendritic cells transport tissue derived antigens from necrotic cells in the heart to local mediastinal lymph nodes, where they cross-present self-antigens on major histocompatibility complex I complexes to CD8^+^ T cells in a stimulatory manner, rather than in a tolerogenic manner.^33^ Support for this possibility was demonstrated in a study wherein blockade of dendritic cell priming in mice lacking the C-type lectin receptor 9A (*Clec9a*^*−/−*^) prevented the activation of cytotoxic CD8^+^ T cells following ISO-induced myocardial injury.^34^ Importantly, upregulation of cell intrinsic PD-L1 on dendritic cells provides an immunosuppressive signal to CD8^+^ T cells, thereby decreasing antigen-specific CD8^+^ T cell proliferation and maintaining self-antigen–specific T cells in a nonresponsive tolerant state.^35^ Accordingly, loss of this restraining inhibitory signal would be expected to switch dendritic cells from being tolerogenic to being immunostimulatory.

The results of the present study both confirm and expand upon the paradigm of self-antigen–mediated expansion of clonal effector CD8^+^ cells proposed by Axelrod et al^28^ and others^36^ in order to explain the development of spontaneous myocarditis in *Pdc1*^−/−^/*Ctla4*^+/−^ mice. The observation in the present study that 3 sequential priming low doses of ISO were necessary but not sufficient for the development of sustained CD8^+^ inflammation in the PD-1^−/−^ mice, coupled with the observation that there were increased numbers of cDCs and CD8^+^ effector memory cells in the mediastinal lymph nodes draining the ISO-injured hearts of PD-1^−/−^ mice but not in WT mice, suggests (but does not prove) that tissue-derived antigen cross-priming of dendritic cells is required for the expansion of self-reactive effector CD8^+^ T cells and that loss of suppression of self-reactive effector CD8^+^ T cell clones by PD-1^−/−^dendritic cells is responsible, at least in part, for the expansion of self-reactive CD8^+^ T cell clones and the development of sustained low-grade myocarditis in the ISO^primed^/ISO^injury^ PD1^−/−^mice. Insofar as we did not perform TCR sequencing, we cannot determine whether the loss of peripheral immune tolerance resulted in the expansion of cardiac-specific protein α-myosin T cell clones, the expansion of self-reactive low-affinity TCR CD8^+^ T cell clones, or the expansion of both subsets of self-reactive CD8^+^ T cells. Additional further studies will be necessary to address this important question, as well as the role of the PD-1/PD-L1 signaling axis with respect to dendritic cell cross-priming of CD8^+^ T lymphocytes following ISO injury.

## Conclusions

The major new finding of this study is that recurrent neurohormonal stress triggers a persistent dysregulated CD8^+^ myocardial inflammatory response in PD-1^−/−^ mice that is characterized by increased cardiac hypertrophy, mild LV dysfunction, and increased lethality. Although direct correlations between short-term observations in PD-1^−/−^ mice and long-term effects in ICI-treated cancer patients are not appropriate, these findings do provide potential insights into the development of smoldering myocarditis that has been reported in some cancer patients treated with ICIs.^11^^,^^12^^,^^37^ First, our findings are consistent with the observation that there is a wide spectrum in the severity of the myocardial inflammatory responses in ICI-treated patients, ranging from mild myocardial inflammation to fulminant myocarditis.^37^ Second, the observation that low levels of repetitive cardiac injury are required for the development of sustained CD8^+^ myocardial inflammatory response in PD-1^−/−^ mice is in accordance with clinical reports that indicate that hypertension and coronary artery disease are frequent comorbidities in the ICI-treated cancer patients that develop smoldering myocarditis,^37^ and raise the possibility that episodic bouts of low levels of myocardial injury (e.g. poorly controlled hypertension or recurrent angina) may be sufficient to lead to the clonal expansion of self-reactive CD8^+^ T cell responses in the hearts of ICI-treated patients. Finally, our findings raise the interesting possibility (albeit speculative) that tolerizing dendritic cells, which increases interleukin-10 levels, may be an effective immunomodulatory strategy for treating patients with ICI-induced myocarditis.^38^^,^^39^

Perspectives **COMPETENCY IN MEDICAL KNOWLEDGE:** Despite the increasing number of cancer patients and survivors treated with ICIs, the mechanisms of ICI-induced myocarditis or functions of immune checkpoints in the heart remain unclear. Here, we provide evidence that recurrent neurohormonal stress triggers a persistent dysregulated CD8^+^ myocardial inflammatory response in PD-1^−/−^ mice that is consistent with the smoldering myocarditis phenotype reported in cancer patients who are being treated with ICIs. These findings raise the possibility that episodic bouts of low levels of myocardial injury (eg, poorly controlled hypertension or recurrent angina) may be sufficient to lead to the clonal expansion of self-reactive CD8^+^ T cell responses in the hearts of ICI-treated patients.

**TRANSLATIONAL OUTLOOK:** Our experimental findings are entirely consistent with the features of smoldering myocarditis that is increasingly being reported in cancer patients receiving ICIs, and expand on the paradigm that self-antigen–mediated expansion of clonal effector CD8^+^ cells is an important mechanism of ICI-induced myocarditis. Our results further suggest that tissue derived antigen cross-priming of dendritic cells is required for expansion of self-reactive effector CD8^+^ T cells, and that loss of suppression of self-reactive effector CD8^+^ T cell clones by PD-1^−/−^dendritic cells is responsible, at least in part, for expansion of self-reactive CD8^+^ T cell clones and the development of sustained low-grade myocarditis in the ISO-injured PD1^−/−^mice. Although speculative, our findings raise the interesting possibility that tolerizing dendritic cells may be an effective immunomodulatory strategy for treating patients with ICI-induced myocarditis.

## Funding Support and Author Disclosures

This study was supported by research funds from the National Institutes of Health (R01HL147968, R01 HL155344, S10 OD028597), Veterans Administration (AN #4345132), and Wilkinson Foundation. Dr Hayashi was supported by grants from the MSD Life Science Foundation, Uehara Memorial Foundation, Mochida Memorial Foundation for Medical and Pharmaceutical Research, and Life Science Foundation of Japan. Dr Lim was supported by a fellowship from the Banting Postdoctoral Fellowships program. All other authors have reported that they have no relationships relevant to the contents of this paper to disclose.
