# Supplementary material for: Recurrent Adrenergic Stress Provokes Persistent Myocarditis in PD-1–Deficient Mice
Source: JACC Basic Transl Sci. 2023 Sep 20;8(12):1503–17. doi: 10.1016/j.jacbts.2023.07.012 (PMC10774592; doi:10.1016/j.jacbts.2023.07.012)
Supplement: Supplemental Table 1 and Supplemental Figures 1-7 [file mmc1.docx]

**Table of Contents for Supplemental Appendix**

| **Supplemental Appendix** | **Page** |
| --- | --- |
| **Supplemental Table S1**. Antibodies used in flow cytometry experiments……….. | 1 |
| **Supplemental Figure S1**. Effects of repetitive low dose isoproterenol injections (ISO^primed^) on cardiac injury and survival in female wild-type and PD-1^-/-^ mice….. | 2 |
| **Supplemental Figure S2**. Gating strategy for the flow cytometric analysis used to identify immune cell populations in the heart ………………………..….......…...... | 3 |
| **Supplemental Figure S3**. Characterization of ISO^primed^/ISO^injury^ female and male PD-1^-/-^ mice ………………………….………………….…………………….…... | 4 |
| **Supplemental Figure S4**. Characterization of ISO^primed^ female PD-1^-/-^ mice treated with phosphate buffered saline or isoproterenol…………..…...…………. | 5 |
| **Supplemental Figure S5**. Gating strategy and flow cytometry analysis of the myocardial dendritic cells in ISO^primed^/ISO^injury^  female wild-type and PD-1^-/-^ mice..….................................................................................................................. | 6 |
| **Supplemental Figure S6**. Gating strategy for the flow cytometric analysis used to identify T cell populations in the mediastinal lymph nodes and spleens………. .… | 6 |
| **Supplemental Figure S7**. Histology of lung, kidney, skeletal muscle and liver in ISO^primed^/ISO^injury^ female PD-1 mice…...….....…................................................. | 7 |

**Supplemental Table S1.** Antibodies used in flow cytometry experiments

| Specificity | Brand | Clone | Fluorophore | Volume | Catalog# |
| --- | --- | --- | --- | --- | --- |
| CD45 | BioLegend | 30-F11 | PerCP/Cy5.5 | 0.5 μl/sample | 103132 |
| CD4 | BioLegend | GK1.5 | PE-Cy7 | 0.5 μl/sample | 100421 |
| CD8a | BD Biosciences | 53-6.7 | BV421 | 0.5 μl/sample | 563898 |
| CD19 | BioLegend | 1D3/CD19 | APC | 0.5 μl/sample | 152410 |
| CD11b | BioLegend | M1/70 | BV510 | 0.5 μl/sample | 101263 |
| Ly6G | BioLegend | 1A8 | FITC | 0.5 μl/sample | 127606 |
| Ly6C | BioLegend | HK1.4 | APC-Cy7 | 0.5 μl/sample | 128025 |
| CD64 | BioLegend | X54-5/7.1 | PE | 0.5 μl/sample | 139303 |
| CD3 | BioLegend | 17A2 | APC | 0.5 μl/sample | 100236 |
| CD44 | BD Biosciences | IM7 | APC-Cy7 | 0.5 μl/sample | 560568 |
| CD62L | BD Biosciences | MEL-14 | FITC | 0.5 μl/sample | 553150 |
| Foxp3 | eBioscience | FJK-16s | APC | 1.0 μl/sample | 17-5773-82 |
| I-A/I-E (MHC class II) | BioLegend | M5/114.15.2 | PE-Cy7 | 0.5 μl/sample | 107630 |
| CD11c | BioLegend | N418 | APC | 0.5 μl/sample | 117310 |
| CD103 | BioLegend | 2E7 | BV421 | 0.5 μl/sample | 121422 |

**
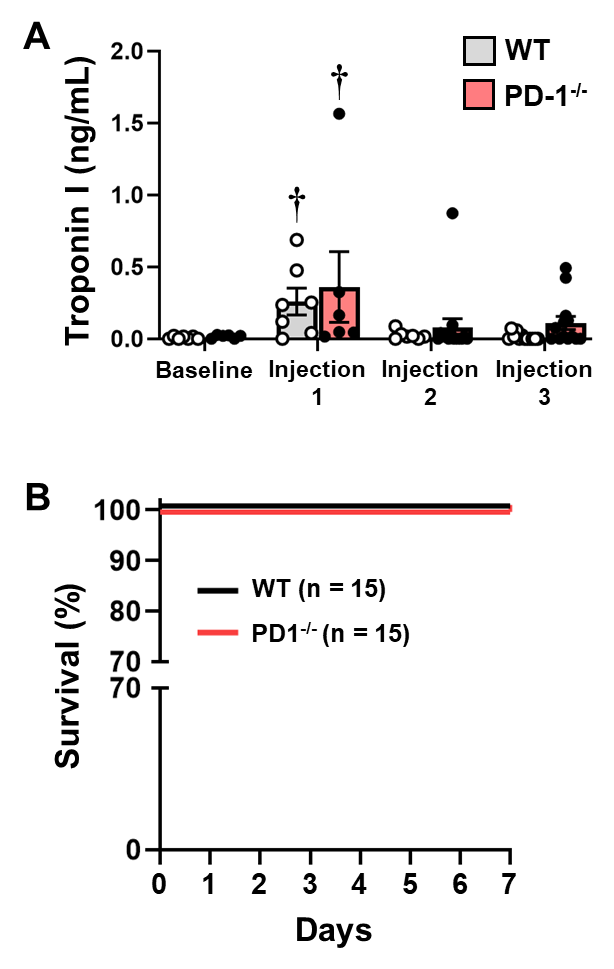
**

**Supplemental Figure S1.** Effects of repetitive low dose isoproterenol (ISO) (100 mg/kg i.p.) injections on cardiac injury and survival in female wild-type (WT) and PD-1^-/-^ mice (referred to as ISO^primed^). **(A)** Serum troponin I levels at baseline and 24 hours after ISO injection on days 0, 2 and 4 (n = 6-14 mice/group/time). The data were taken from a prior publication in order to reduce the number of mice sacrificed (JACC Basic Transl Sci 2022;7 1120 – 1139). **(B)** Kaplan-Meier survival curves of ISO^primed^ WT and PD-1^-/-^ mice. Data were analyzed by repeated measures one-way analysis of variance with Dunnett post-hoc test relative to baseline within groups and repeated measures two-way analysis of variance with Sidak post-hoc test between groups **(A)**. † *P* < 0.05 relative to baseline.


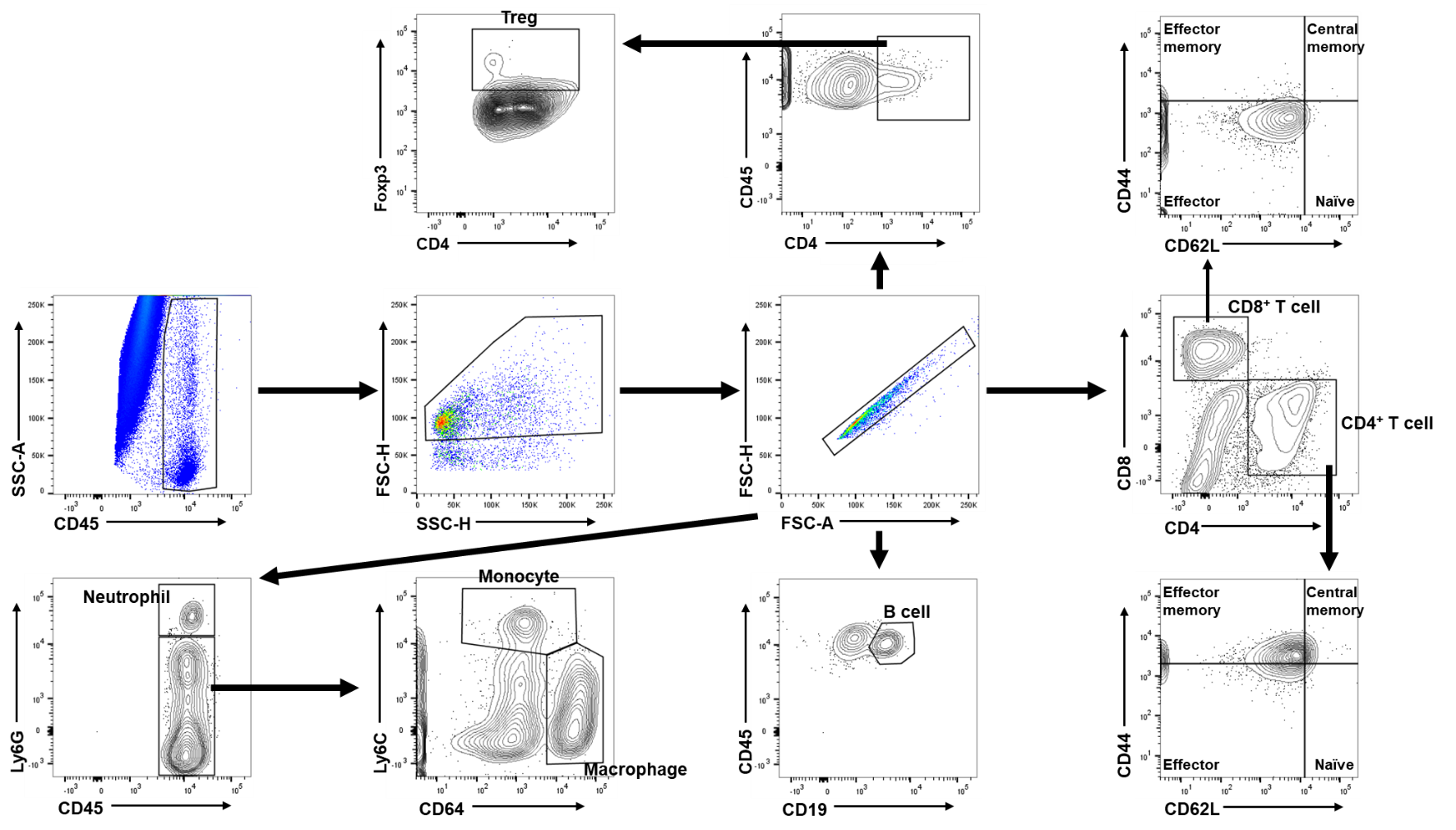


**Supplemental Figure S2.** Gating strategy for the flow cytometric analysis used to identify immune cell populations in the heart.


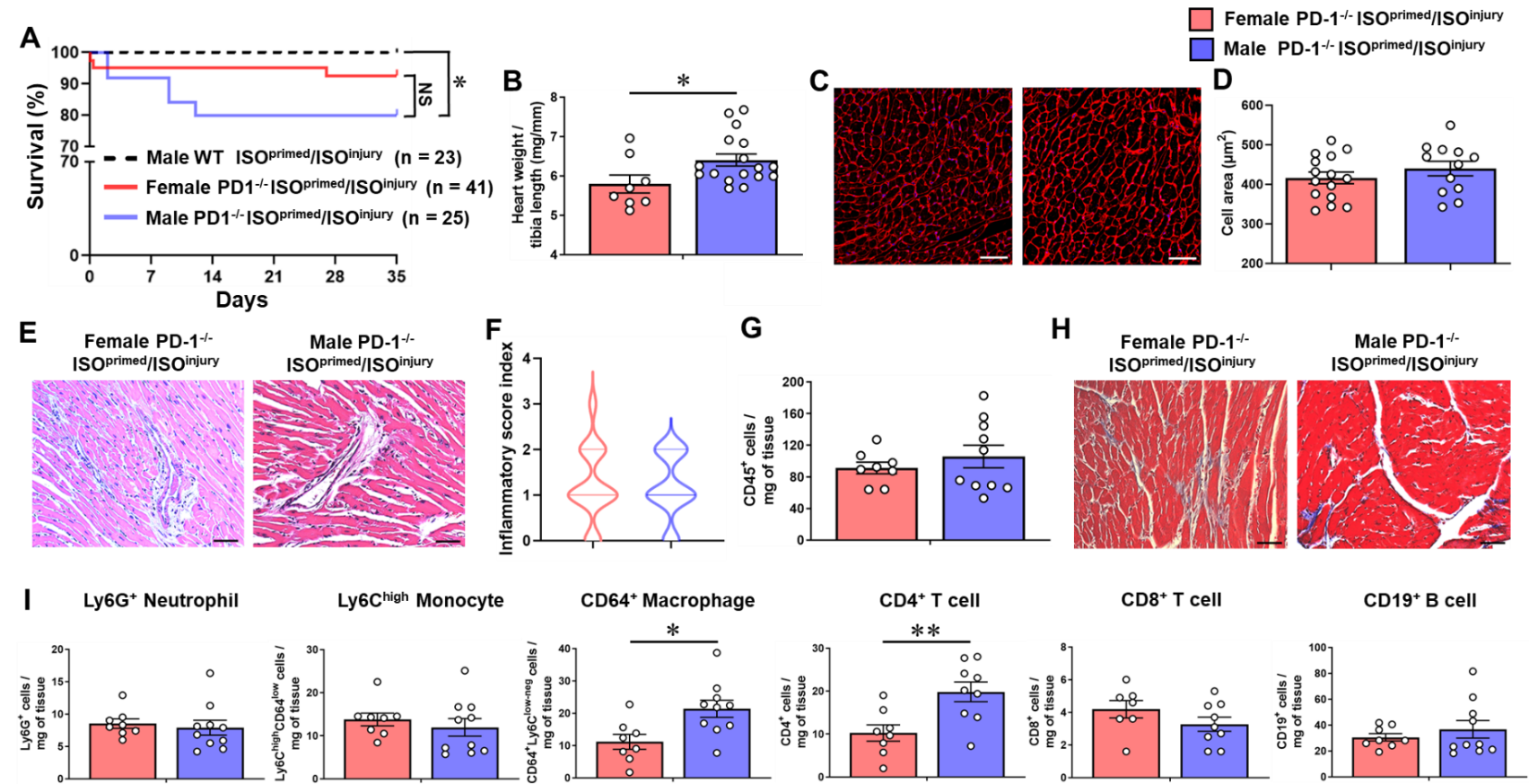


**Supplemental Figure S3.** **(A)** Kaplan-Meier survival curves of ISO^primed^/ISO^injury^ female and male PD-1^-/-^ mice and ISO^primed^/ISO^injury^ male WT mice **(B)** Heart weight-to-tibia length ratios in (n = 8-17 mice/group) of ISO^primed^/ISO^injury^ female and male PD-1^-/-^ mice on day 35 **(C)** Representative photographs of wheat germ agglutinin (WGA) staining (scale bar = 50 µm) in ISO^primed^/ISO^injury^ female and male PD-1^-/-^ mice on day 35 **(D)** Group data of cardiac myocyte cell area based on WGA staining in ISO^primed^/ISO^injury^ female and male PD-1^-/-^ mice on day 35 (n = 20 cells counted/section, 3 sections/heart, 4-5 mouse hearts/group). **(E)** Representative photographs of hematoxylin and eosin (H&E)-stained hearts of ISO^primed^/ISO^injury^ female and male PD-1^-/-^ mice on day 35 (scale bar = 50 µm). **(F)** Inflammatory score index based on H&E staining in ISO^primed^/ISO^injury^ female and male PD-1^-/-^ mice on day 35 (n = 24-30 fields obtained from 4-5 hearts/group/time). **(G)** Flow cytometry analysis of the number of CD45^+^ cells/mg of heart tissue in ISO^primed^/ISO^injury^ female and male PD-1^-/-^ mice on day 35 (n = 8-10 hearts/group). **(H)** Representative photographs of Masson’s trichrome-stained hearts ISO^primed^/ISO^injury^ female and male PD-1^-/-^ mice on day 35 **(**scale bar = 50 µm). **(I)** Flow cytometry analysis of the number of myocardial immune cell subsets among CD45^+^ cells (cells/mg of tissue) in ISO^primed^/ISO^injury^ female and male PD-1^-/-^ mice on day 35 (n = 7-10 hearts/group). Data in panels B, D, F, G, and I were analyzed by two-tailed Student's *t*-test. **P* < 0.05, ***P* < 0.01.


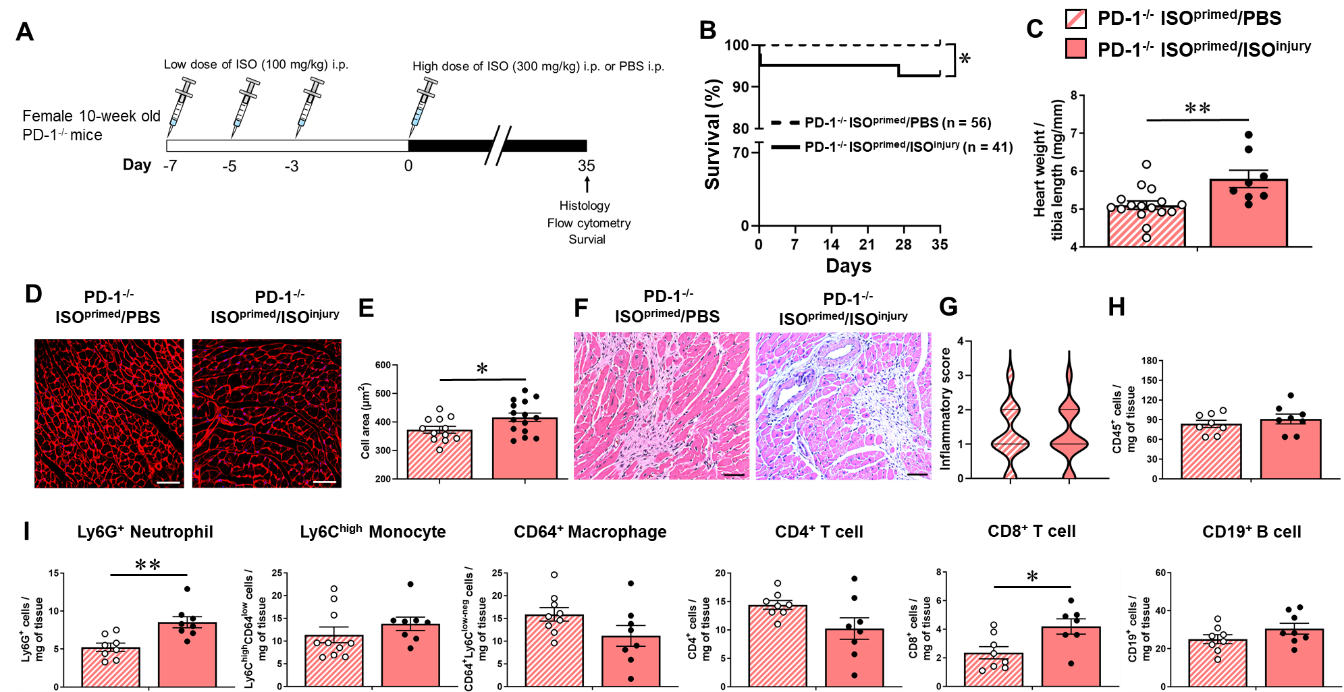


**Supplemental Figure S4.** Characterization of ISO^primed^ female PD-1^-/-^ mouse hearts treated i.p. with phosphate buffered saline (ISO^primed^/PBS) or isoproterenol (ISO^primed^/ISO^injury^) at baseline (day 0) and then followed through 35 days. **(A)** Outline Study protocol. **(B)** Kaplan-Meier survival curves **(C)** Heart weight-to-tibia length ratios (n = 8-16 mice/group). **(D)** Representative photographs of wheat germ agglutinin (WGA) staining (scale bar = 50 µm). **(E)** Group data of cardiac myocyte cell area based on WGA staining (n = 20 cells counted/section, 3 sections/heart, 4-5 mouse hearts/group). **(F)** Representative photographs of hematoxylin and eosin (H&E)-stained hearts (scale bar = 50 µm). **(G)** Inflammatory score index based on H&E staining (n = 24-30 fields obtained from 4-5 hearts/group/time). **(H)** Flow cytometry analysis of the number of CD45^+^ cells/mg of heart tissue (n = 8 hearts/group). **(I)** Flow cytometry analysis of the number of myocardial immune cell subsets among CD45^+^ cells (cells/mg of tissue) (n = 8-10 hearts/group). Data in panels C, E, G, H, and I were analyzed by two-tailed Student's *t*-test. **P* < 0.05, ***P* < 0.01.


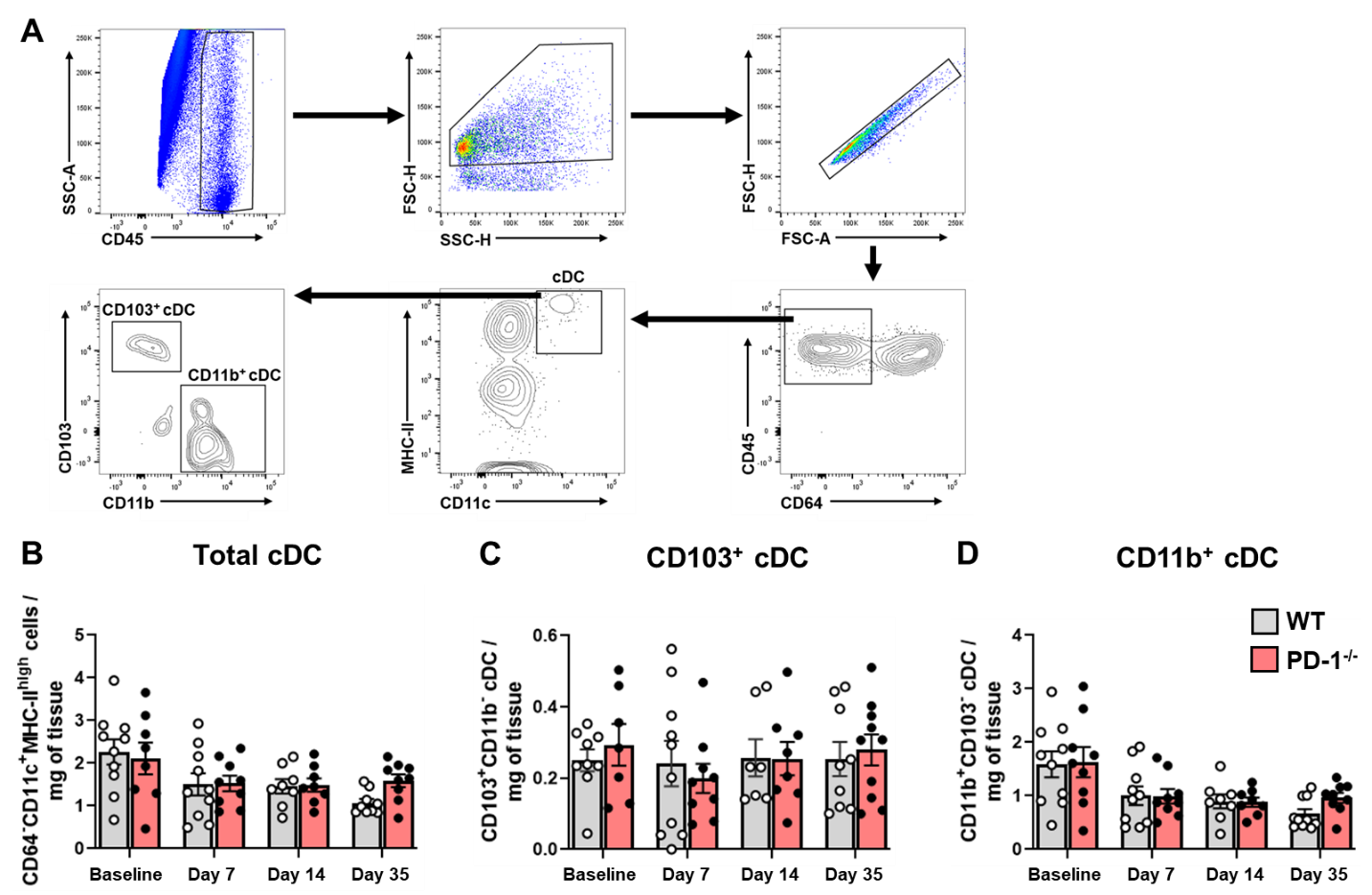


**Supplemental Figure S5.** Flow cytometry analysis of the numbers of myocardial dendritic cells (DCs)/mg of tissue ISO^primed^/ISO^injury^ female WT and PD-1^-/-^ mice. **(A)** Gating strategy used. **(B)** Total number of conventional DCs (cDCs)/mg tissue identified as CD64^-^CD11c^+^MHC-II^high^ cells, **(C)** CD103^+^ cDCs, and **(D)** CD11b^+^ cDCs (n = 7-10 mice/group/time). Data were analyzed by repeated measures two-way analysis of variance with Sidak post-hoc test.


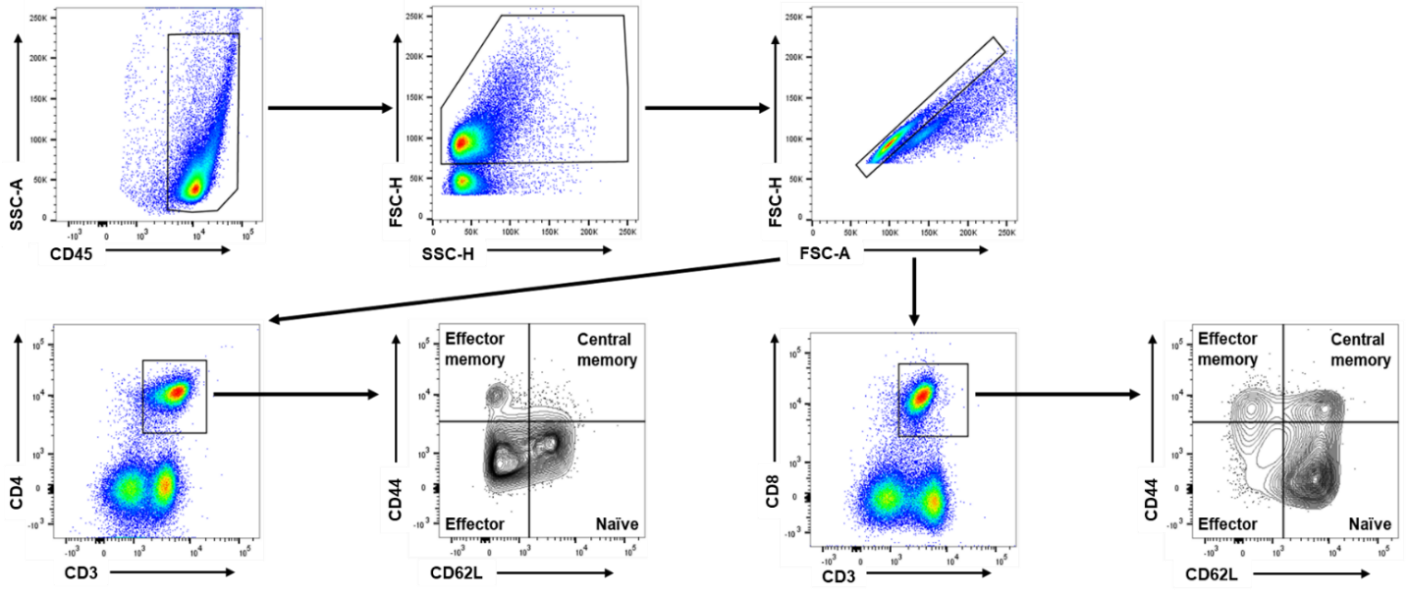


**Supplemental Figure S6.** Gating strategy for the flow cytometric analysis used to identify subsets of T cell populations in the mediastinal lymph nodes and spleens.


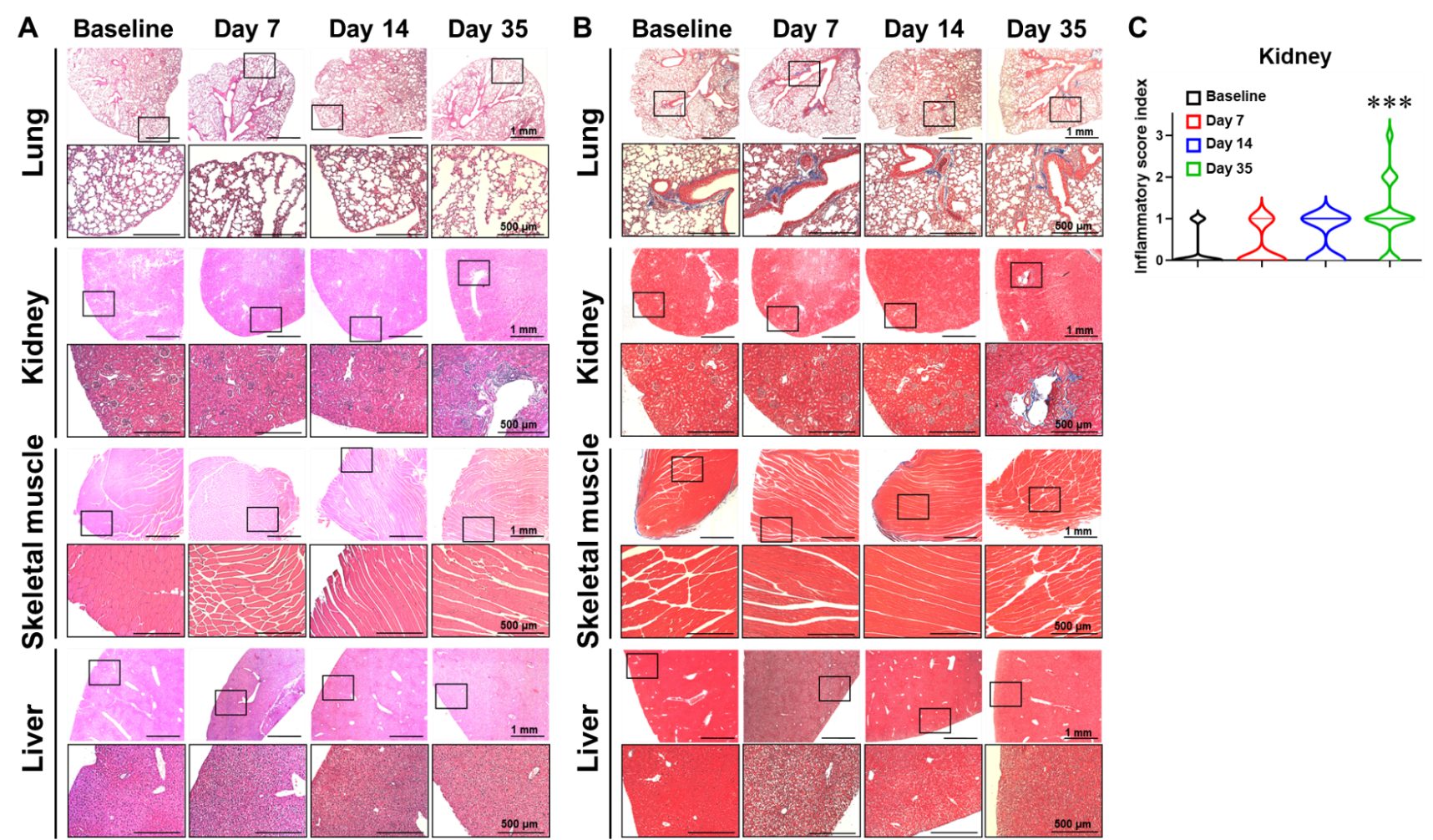


**Supplemental Figure S7**.  **(A)** Representative photographs of hematoxylin and eosin-stained sections of lungs, kidney, skeletal muscle and liver in ISO^primed^/ISO^injury^ female PD-1^-/-^ mice at baseline and 7, 14, and 35 days. Figures in the upper panels are shown at low magnification (2.5X) (scale bar = 1 mm) and the lower panels depict high-power fields of the cropped images shown in the upper panels (10X) (scale bar = 500 µm). **(B)** Representative photographs of Masson’s trichrome-stained photographs of lungs, kidney, skeletal muscle and liver in ISO^primed^/ISO^injury^ female PD-1^-/-^ mice at baseline and 7, 14, and 35 days. Figures in the upper panels are shown at low magnification (2.5X) (scale bar = 1 mm) and the lower panels depict high-power fields of the cropped images shown in the upper panels (10X) (scale bar = 500 µm). **(C)** Inflammatory score index of the kidney ISO^primed^/ISO^injury^ female PD-1^-/-^ mice at baseline and 7, 14, and 35 (n = 18 fields obtained from 3 kidneys/time). The degree of inflammation in the kidney was scored semi-quantitatively using an inflammatory score index: 0 = no infiltrate; 1+ = infiltrates involving <25% of the kidney; 2+ = infiltrates involving 25%-50% of the kidney; 3+ = infiltrates involving 50%-75% of the kidney; and 4+ = infiltrates involving 75%-100% of the kidney. Data were analyzed by repeated measures one-way analysis of variance with Dunnett post-hoc test relative to baseline. ****P* < 0.001.
